# Supramolecular Phenylalanine-Derived Hydrogels for the Sustained Release of Functional Proteins

**DOI:** 10.1021/acsbiomaterials.2c01299

**Published:** 2023-01-24

**Authors:** Melissa L. Jagrosse, Pamela Agredo, Brittany L. Abraham, Ethan S. Toriki, Bradley L. Nilsson

**Affiliations:** † Department of Chemistry, University of Rochester, Rochester, New York14627, United States; ‡ Materials Science Program, University of Rochester, Rochester, New York14627, United States

**Keywords:** hydrogel, controlled release, self-assembly, drug delivery

## Abstract

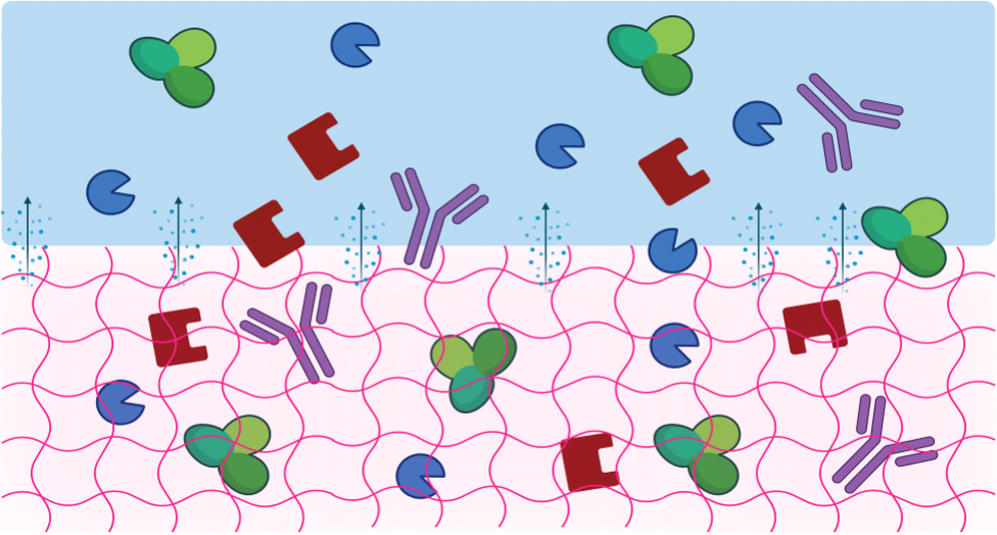

Protein-based therapeutics have emerged as next-generation pharmaceutical agents for oncology, bone regeneration, autoimmune disorders, viral infections, and other diseases. The clinical application of protein therapeutics has been impeded by pharmacokinetic and pharmacodynamic challenges including off-target toxicity, rapid clearance, and drug stability. Strategies for the localized and sustained delivery of protein therapeutics have shown promise in addressing these challenges. Hydrogels are critical materials that enable these delivery strategies. Supramolecular hydrogels composed of self-assembled materials have demonstrated biocompatibility advantages over polymer hydrogels, with peptide and protein-based gels showing strong potential. However, cost is a significant drawback of peptide-based supramolecular hydrogels. Supramolecular hydrogels composed of inexpensive low-molecular-weight (LMW) gelators, including modified amino acid derivatives, have been reported as viable alternatives to peptide-based materials. Herein, we report the encapsulation and release of proteins from supramolecular hydrogels composed of perfluorinated fluorenylmethyloxcarbonyl-modified phenylalanine (Fmoc-F_5_-Phe-DAP). Specifically, we demonstrate release of four model proteins (ribonuclease A (RNase A), trypsin inhibitor (TI), bovine serum albumin (BSA), and human immunoglobulin G (IgG)) from these hydrogels. The emergent viscoelastic properties of these materials are characterized, and the functional and time-dependent release of proteins from the hydrogels is demonstrated. In addition, it is shown that the properties of the aqueous solution used for hydrogel formulation have a significant influence on the *in vitro* release profiles, as a function of the isoelectric point and molecular weight of the protein payloads. These studies collectively validate that this class of supramolecular LMW hydrogel possesses the requisite properties for the sustained and localized release of protein therapeutics.

## Introduction

Protein-based therapeutics have emerged as promising agents for oncology,^1,2^ bone regeneration,^3^ autoimmune disorders, viral infections, and other diseases.^4−6^ Protein-based therapeutics have various modes of action including replacement or supplementation of deficient/abnormal proteins, augmentation of or interference with signaling pathways, provision of novel function and/or activity, and delivery of appended payloads such as radionuclides, cytotoxic drugs, or protein effectors.^7^ Although many small-molecule drugs have been developed to combat diseases, proteins are advantageous in a variety of ways, including increased specificity in binding interactions and the ability to modify existing proteins via protein engineering strategies to generate novel functionalities.^8^ Protein therapeutics are often, but not always, well tolerated *in vivo* with minimal off-target effects.^7,9^ Advances in protein expression and production have enabled the development of proteins as critical next-generation therapeutics.

*In vivo* delivery and administration of protein therapeutics presents a significant barrier that has challenged the practical application of these agents. Protein-based therapeutics are largely limited to cell-surface receptor targets, due to the inability of large proteins to cross the cell membrane.^7^ Oral administration of protein-based therapies is impractical due to protein degradation by digestive enzymes and extremely acidic gastric environment.^7^ Administration via other routes is likewise hindered by susceptibility to proteolytic degradation, resulting in short serum half-lives of protein therapeutics *in vivo*. In some cases, protein therapies have elicited strong immune responses due to a variety of factors including post-translational modifications, impurities maintained through the drug-making process, and the propensity of expressed proteins to undergo aggregation.^10^ The challenges associated with the delivery of protein biologics have led to the development of delivery systems that enable the sustained and localized release of protein payloads. Ideal protein delivery systems protect and maintain *in vivo* stability of protein payloads, minimize off-target effects, and reduce dose and/or dosing frequency.^7^

Hydrogels have emerged as critical biomaterials for the localized and sustained release of small molecules and biomacromolecule therapeutics.^11^ Ideal hydrogel materials for *in vivo* drug delivery should be shear-responsive, which enables nonsurgical delivery by injection. They should also maintain integrity over periods of days to weeks in bodily fluids and tissues to enable gradual release of encapsulated payloads over time and should be nonirritating and nonimmunogenic.^7^ Polymer-based hydrogels have been developed for the sustained release of proteins including insulin,^12^ lysozyme,^13,14^ α-chymotrypsin,^15,16^ bovine serum albumin (BSA),^14,17,18^ vascular endothelial growth factor (VEGF),^19^ platelet-derived growth factor B (PDGFB),^19,20^ and monoclonal antibodies.^21^ Unfortunately, some polymer-based hydrogels have been shown to be cytotoxic upon degradation *in vivo* and are often nonbiocompatible due to immunogenic responses to the materials.^22^ For this reason, hydrogels composed of supramolecular assemblies of peptides, proteins, carbohydrates,^23^ and oligonucleotides have been employed as biocompatible alternatives to polymer hydrogels.

Peptide-derived self-assembled hydrogels have been particularly effective for drug delivery applications, including the delivery of protein payloads.^24−26^ Koutsopoulos et al. demonstrated that supramolecular hydrogels formulated from the self-assembled Ac-(RADA)_4_-NH_2_ peptide facilitated the controlled release of lysozyme, trypsin inhibitor (TI), BSA, and immunoglobulin G (IgG).^27^ They found that the rate of protein release from these neutral hydrogels decreased with increasing molecular weight of the payload, and that released proteins maintained functional catalytic/binding activity. Schneider and co-workers demonstrated that supramolecular peptide hydrogels composed of the positively charged VLTKVKTKV^D^P^L^PTKVEVKVLV-NH_2_ (HLT2) or the negatively charged VEVQVEVEV^D^P^L^PTEVQVEVEV-NH_2_ (VEQ3) peptides effectively released α-lactalbumin (14.1 kDa, pI 4.2–4.5), myoglobin (14.7 kDa, pI 7.0), or lactoferrin (77 kDa, pI 8.4–9).^28^ Proteins encapsulated in hydrogels of like charge demonstrated over 80% release after 4 days, while proteins encapsulated in hydrogels of opposite charge were largely retained in the network. Neutral myoglobin was released to similar degrees in the HLT2 (90%) and VEQ3 (95%) hydrogels.

**Figure 1:**
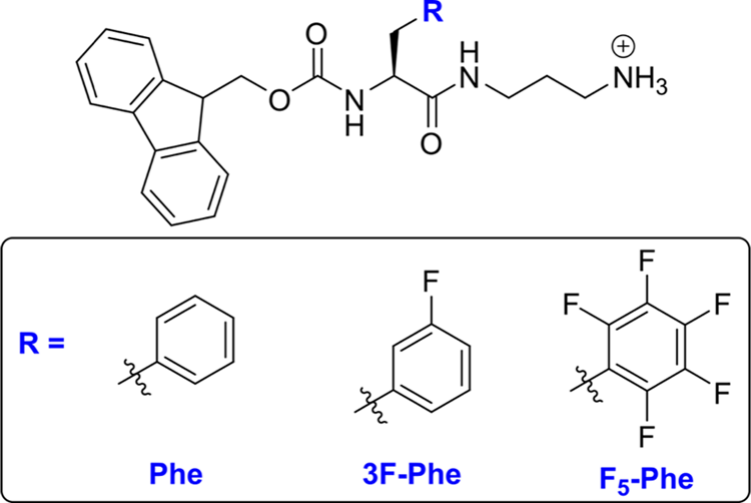
Chemical structures of Fmoc and diaminopropane (DAP) phenylalanine (Phe) derivatives that form self-assembled hydrogels for drug delivery applications.

The widespread adoption of supramolecular peptide-based hydrogels for protein release has been impeded due to the relatively high cost of peptide synthesis and purification, resulting in hydrogels that are often more expensive to produce than the therapeutic cargo.^29^ Supramolecular hydrogels composed of low-molecular-weight (LMW) materials, including dipeptides and functionalized amino acids, have been recently developed as alternatives to peptide assemblies.^30−33^*N*-Fluorenylmethyloxycarbonyl phenylalanine (Fmoc-Phe) derivatives are a privileged class of molecule that form self-assembled hydrogels that have the requisite properties for drug delivery applications.^31,34,35^ Recently, we reported cationic Fmoc-Phe derivatives that spontaneously form hydrogel networks (Figure 1)^36^ and validated sustained *in vivo* delivery of the anti-inflammatory drug, diclofenac, from these materials for functional pain remediation in mice lasting nearly two weeks.^37^ We also determined that the release rate of small-molecule cargo from these cationic hydrogels was strongly correlated to the charge of cargo molecules, with positive and neutral molecules released rapidly, while negative molecules were highly retained within the network.^38^ This work is in agreement with a recent report of dye release from cationic peptide supramolecular hydrogels by Schneider and co-workers.^28^ Validation of LMW hydrogels for the release of larger biomacromolecules, like proteins, would provide next-generation materials that address some of the limitations of current delivery vectors for the localized, sustained release of protein therapeutics.

Accordingly, in the present study, we characterize the release of four model proteins (RNase A, trypsin inhibitor, BSA, and human IgG, Table 1) from cationic Fmoc-F_5_-Phe-DAP LMW supramolecular hydrogels. The hydrogels were formulated under two conditions, resulting in protein-loaded materials that are at neutral (pH ≈ 7) or slightly acidic pH (pH ≈ 5). The fibril network structures of the Fmoc-F_5_-Phe-DAP hydrogels remain unchanged when loaded with proteins, regardless of gelation method. However, gelation method and the identity of the protein loaded do impact hydrogel viscoelasticity. Protein release profiles strongly depend on the acidity of the hydrogel network. When the protein isoelectric point is near the pH of the hydrogels, release is mainly dependent on the molecular weight of the protein. However, release becomes increasingly dependent on charge as the pH diverges from the isoelectric point of the protein cargo. Finally, the released proteins maintain native fold and function. Collectively, these results validate that Fmoc-Phe-derived hydrogels are inexpensive materials that possess the necessary features for sustained and localized delivery of protein therapeutics.

**Table 1:** Model Proteins Released from Fmoc-F_5_-Phe-DAP Hydrogels

protein	abbreviation	MW (kDa)	isoelectric point (pI)
ribonuclease A	RNase A	13.7^39^	9.6^40^
trypsin inhibitor	TI	20.1^41^	4.5^42^
bovine serum albumin	BSA	66.43^43^	4.5–5.0
human immunoglobulin G	IgG	150^44−46^	6.9^47^

## Materials and Methods

### Materials

Fmoc-F_5_-Phe-DAP was prepared by previously reported methods.^36^ Proteins were purchased from Sigma-Aldrich (RNase A #101091690; BSA #A9647), Fisher Scientific (Trypsin Inhibitor #J60982), or BioFront Technologies (Human IgG, HU-IGG-1). All other reagents and solvents were purchased from commercial vendors and used without further purification. Vendors for specific reagents are listed in the following sections where appropriate.

### Hydrogelation Conditions

Cationic Fmoc-F_5_-Phe-DAP (4.3 mg) was dissolved in 375 μL of deionized water using heat (70 °C) and sonication. After the solution was cooled to room temperature, 25 μL of protein solution in water (20 mg mL^–1^) was added. To trigger gelation, 100 μL of NaCl (570 mM) prepared in deionized water or 100 μL 1× Dulbecco’s modified Eagle’s medium (DMEM) was added to the solutions and quickly mixed with a pipet. The gels were then briefly centrifuged to generate a flat surface and incubated for 1 h at 37 °C. The final gels contained 15 mM Fmoc-F_5_-Phe-DAP, 114 mM NaCl, and 1 mg mL^–1^ protein (pH ∼ 5) or 15 mM Fmoc-F_5_-Phe-DAP, 33 mM DMEM, and 1 mg mL^–1^ protein (pH ∼ 7).

### Oscillatory Rheology

Oscillatory rheology was conducted using a TA Instruments Discovery HR-2 rheometer operating in oscillatory mode. A 20 mM parallel-plate geometry and standard Peltier plate were used for the experiments. Hydrogels of 1 mL volume were formed in 1.5 mL plastic microcentrifuge tubes following the assembly procedure previously described and allowed to stand for 24 h. The gap was set individually for each experiment with an average gap size of 1.2 mm. To determine the linear viscoelastic region for each sample, strain sweeps were performed from 0.01 to 100% strain at a constant angular frequency of 1 Hz (6.28 rad s^–1^) (Figures S1 and S2, Supporting Information). Before measurement of frequency sweep, a time sweep at constant strain of 0.2% and constant angular frequency of 0.1 rad s^–1^ was performed for 300 s to allow the sample to equilibrate on the plate after the transfer process. Then, frequency sweep experiments for each sample were performed from 0.1 to 100 rad s^–1^ at a constant strain of 0.2%, which was within the determined linear viscoelastic region for all hydrogels studied. Values at the upper end of the frequency sweep were cut off from reported data when the raw phase angle increased above 175° as recommended for the TA DHR series of rheometers since values beyond this point are dominated by the instrument inertial torque instead of the sample torque.^48^ Reported values for storage moduli (*G*′) and loss moduli (*G*″) are the average of at least three distinct measurements on separate hydrogels with the error reported as the standard deviation about the mean.

The average storage moduli (*G*′) of each system was used to calculate the mesh size (ξ) using eq 1, where *G*′ is the average storage modulus, *k*_B_ is the Boltzmann constant, and *T* is the temperature.^49,50^
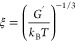
1

### Transmission Electron Microscopy (TEM)

Aliquots of assembled materials (5 μL) were applied directly onto 200 mesh carbon-coated copper grids and allowed to stand for 1 min. Excess sample was carefully removed by capillary action using filter paper, and the grids were then stained with 2% (w/v) uranyl acetate (5 μL) for 10 min. Excess stain was removed by capillary action, and the grids were allowed to air-dry for 5 min. TEM images were taken using a Hitachi 7650 transmission electron microscope with an accelerating voltage of 80 kV. Dimensions of the network structures were determined using ImageJ software and are reported as the average of at least 100 independent measurements with error reported as the standard deviation about the mean.

### Protein Release Profiles

Cationic Fmoc-F_5_-Phe-DAP hydrogels containing various proteins were prepared as described above to form a 500 μL hydrogel containing 15 mM gelator, 114 mM NaCl, and 1 mg mL^–1^ protein. A solution of 1× phosphate-buffered saline (PBS) was prepared by 1:10 dilution of a 10× PBS solution (Corning 46-013-CM, pH 7.4). The resulting 1× PBS was slowly laid over the gel, and this two-phase gel/solution mixture was sealed in a vial and incubated at 37 °C. Aliquots of buffer solution (100 μL) were removed at 1, 4, 8, 24, 48, and 72 h from the initial layering of PBS over the hydrogel. After removing each aliquot, the buffer solution was immediately replenished by an equal volume of PBS (pH 7.4, 100 μL). For all experiments, protein concentration was determined by correlation to a high-performance liquid chromatography (HPLC) standard curve.^51^ The standard curve for each protein was constructed by a serial dilution of the native protein and injection onto a Shimadzu 2010A analytical HPLC equipped with a Phenomenex Gemini column (5 μm, C18, 250 × 4.6 nm). A gradient of water and acetonitrile (0.05% TFA) was used as the mobile phase eluent at a flow rate of 1 mL min^–1^ and UV detection was monitored at 215 nm (see Supporting Information Table S1 for HPLC mobile phase conditions, Figures S3–S6 for analytical HPLC traces, and Figures S7–S10 for HPLC concentration curves). Absolute protein concentrations for the standard curve were determined by amino acid analysis (UC–Davis, Davis, CA).^52^

This enabled interpolation of the amount of protein released into the 1.0 mL solution at each timepoint via conversion of the protein concentration to nmol of protein and the diffusion constant was determined using the following equation, where *M*_*t*_/*M*_∞_ is the ratio of molecules of protein released to the total molecules of protein loaded in the system, *t* is the time (min), λ is the gel thickness (height, m), and *D* is the diffusion coefficient (m^2^ min^–1^)^53,54^ (eq 2)
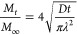
2For each timepoint, the concentration of protein in the aliquot was used to calculate the total amount of protein (nmol) in the 1.0 mL PBS layer. For the 1 h timepoint, this value was used without further manipulation as *M*_*t*_; however, for the remaining timepoints, the amount calculated for *M*_*t*_ was manipulated to include the amount of protein removed in prior aliquots. This manipulation is required so that *M*_*t*_ reflects the total amount of protein released from time zero to time *t*. Data were collected in triplicate and plotted as *M*_*t*_/*M*_∞_ against time (min) with error reported as the standard error of the mean (Figure 4A,B).

To determine the diffusion coefficient, *D*, a second plot was generated by plotting *M*_*t*_/*M*_∞_ against *t*^1/2^ (min^1/2^) from the linear section of the first plot (first 480 min) (Figure 4C,D). The diffusion coefficient, D (m^2^ min^–1^), was determined by measuring the slope of *M*_*t*_/*M*_∞_ against *t*^1/2^ (min^1/2^) and setting this value equal to the coefficient of *t*^1/2^ (min^1/2^) in eq 3
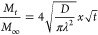
3

### Sodium Dodecyl Sulfate-Polyacrylamide Gel Electrophoresis (SDS-PAGE) and Western Blot of Released Proteins

For the preparation of released protein SDS-PAGE samples, aliquots of gel supernatant or native proteins (10 μL) were mixed with 2× Laemelli buffer (9.5 μL) and β-mercaptoethanol (0.5 μL) and heated to 90 °C for 10 min. RNase A and Trypsin inhibitor samples were loaded onto acrylamide/bis-acrylamide gels consisting of 4% stacking gels and 20% separating gels (4/20%). For BSA and human IgG, samples were loaded onto 4/15% acrylamide/bis-acrylamide gels. SDS-PAGE was performed in 1× running buffer for 60 min at 200 V. Human IgG was run under denaturing and nondenaturing conditions (absence of β-mercaptoethanol) to verify no changes to the secondary structure. RNase A and human IgG gels were imaged on a Bio-R ChemiDoc MS imaging system. TI and BSA gels were washed with dH_2_O and equilibrated in 1× Towbin buffer for 15 min. Proteins were transferred for 1 h at 100 V to nitrocellulose membranes. Membranes were then washed with 0.3% TBS-T three times for 20 min at room temperature followed by dH_2_O for 2 min. Membranes were incubated with Colloidal Gold Protein Stain for approximately 10 min and imaged on a Bio-Rad ChemiDoc MS imaging system.

### RNase A cCMP Hydrolysis Assay^55^

Cytidine 3′,5′-cyclic monophosphate (cCMP) was prepared in assay buffer (20 mM bis-Tris, 1 mM EDTA, pH 8.0). Dilutions of cCMP (500 μL) were combined with native RNase A (500 μL, 1.0 μM) or released RNase A (500 μL; 0.25 μM) in a quartz cuvette. Absorbance (295 nm) was recorded every 0.4 seconds for 10 min on a Shimadzu UV-2401 PC Spectrophotometer. The concentration of CMP was calculated using the Beer–Lambert law (ε_296_ = 0.00038 M^–1^ cm^–1^)^55^ to generate a graph of enzyme velocity (M min^–1^) vs substrate concentration (M) for native and released RNase A. *K*_m_ (M), *V*_max_ (M min^–1^), and *k*_cat_ (s^–1^) values were calculated from a nonlinear regression using Michaelis–Menten kinetics in GraphPad Prism. Catalytic efficiency (*k*_cat_/*K*_m_; M^–1^ s^–1^) was calculated from these values.

### BSA Esterase Assay^56^

*p*-Nitrophenyl acetate (10 mM) was dissolved in dimethyl sulfoxide (DMSO). Seven serial dilutions from the DMSO stock were prepared in assay buffer (20 mM Tris, pH 8). Native or released BSA (10 μM) was mixed with *p*-nitrophenyl acetate and absorbance (401 nm) was recorded every 0.4 seconds for 10 min on a Shimadzu UV-2401 PC Spectrophotometer. The concentration of 4-nitrophenol was calculated using the Beer–Lambert law (ε_401_ = 18,750 M^–1^ cm^–1^)^57,58^ to generate a graph of enzyme velocity (M min^–1^) vs substrate concentration (M) for native and released BSA. A nonlinear regression of the graph using Michaelis–Menten kinetics in GraphPad Prism was used to determine *K*_m_ (M), *V*_max_ (M min^–1^), and *k*_cat_ (s^–1^) values. Catalytic efficiency (*k*_cat_/*K*_m_; M^–1^ s^–1^) was then calculated from these values.

### Trypsin Inhibitor Assay^59,60^

The activity of native TCPK trypsin when exposed to native trypsin inhibitor (TI) and trypsin inhibitor released from our hydrogels was measured using a Pierce Fluorescent Protease Kit. Components of the kit (native TCPK trypsin, assay buffer, and fluorescein isothiocyanate, FITC-casein substrate) were prepared according to the kit instruction manual. TCPK trypsin (500 ng mL^–1^) was dissolved in assay buffer and loaded into a black-bottom Corning 384-well plate (50 μL per well). The FITC-casein substrate (50 μL, 10 μg mL^–1^) was added to each well and fluorescence intensity was measured every 5 min for 45 min on a Tecan Plate Reader. To determine trypsin activity in the presence of TI, native trypsin (500 ng mL^–1^) was incubated with native TI (500 ng mL^–1^) or supernatant from gels loaded with TI for 30 min at room temperature. Samples and controls (50 μL) were loaded into a 384-well plate in triplicate, followed by the addition of FITC-casein (50 μL, 10 μg mL^–1^) to each well and fluorescence intensity was measured every 5 min for 45 min. The average of the blank wells (FITC-casein alone) was subtracted from the fluorescence intensity of the controls and samples. The resulting change in fluorescence intensity (ΔRFU) was plotted against time (min). Additionally, ΔRFU for the controls and samples at 45 min were plotted and analyzed using Brown-Forsythe and Welch analysis of variance (ANOVA) tests in GraphPad Prism.

### Human IgG Sandwich ELISA

The structural integrity of released human IgG was verified using an Invitrogen IgG (Total) Human ELISA kit (Fisher Scientific #88-50550-22). A 96-well plate was coated with a 1:250 dilution of the capture antibody in 1× coating buffer (100 μL) overnight at 4 °C. The wells were aspirated and washed with wash buffer (400 μL) twice, allowing for 1 min of soaking time. Wells were blocked with 1× blocking buffer (250 μL) overnight at 4 °C. Wells were then aspirated and washed with wash buffer (400 μL) twice, allowing for 1 min of soaking time. Serial dilutions of the human IgG standard were prepared in 1× Assay Buffer A (350 μL), in addition to a 1:1 dilution of released Human IgG in 1× Assay Buffer A (350 μL). Samples, controls, and standards were added to the wells in triplicate (100 μL) and incubated for 2 h at room temperature with shaking. The wells were aspirated and washed with wash buffer (400 μL) for time, allowing for 1 min of soaking time. A 1:250 dilution of the capture antibody in 1× Assay Buffer A (100 μL) was added to the appropriate wells and incubated for 1 h at room temperature with shaking. The wells were aspirated and washed with wash buffer (400 μL) four times, allowing for 1 min of soaking time. Substrate (100 μL) was added to the wells and incubated for 15 min at room temperature with shaking, followed by the addition of Stop Solution (2M H_2_PO_4_, 100 μL). Absorbance was measured at 570 and 450 nm. A standard curve was generated from the serial dilutions of the Human IgG standard and fitted using a nonlinear regression in GraphPad Prism (Binding-Saturation One Site Total). Concentrations of Human IgG of controls and samples were interpolated from this standard curve and analyzed using ordinary one-way ANOVA tests.

## Results and Discussion

### Emergent Properties of Protein-Loaded Hydrogels

Hydrogels of Fmoc-F_5_-Phe-DAP (15 mM) loaded with our proteins (1 mg mL^–1^) were prepared using two formulation methods. First, gelation was initiated by the addition of NaCl (100 μL, 570 mM) to a solution of gelator with protein (400 μL, 18.75 mM Fmoc-F_5_-Phe-DAP, 1.25 mg mL^–1^ protein) to provide hydrogels with 15 mM Fmoc-F_5_-Phe-DAP, 114 mM NaCl, and 1 mg mL^–1^ protein. The NaCl solution promotes gelation by screening repulsive charge interactions of the cationic gelator, thus facilitating self-assembly and hydrogel network formation.^36,38,61^ Second, hydrogels were formed by dilution of solutions of gelator with protein (400 μL, 18.75 mM Fmoc-F_5_-Phe-DAP, 1.25 mg mL^–1^ protein) with Dulbecco’s modified Eagle’s medium (DMEM) cell culture media (100 μL, 165 mM ion concentration) to provide hydrogels with 15 mM Fmoc-F_5_-Phe-DAP, ∼33 mM ion concentration, and 1 mg mL^–1^ protein. DMEM contains calcium chloride, sodium chloride, sodium phosphate, sodium bicarbonate, and a mixture of amino acids and vitamins.^62^ It is of sufficient ionic strength to promote self-assembly and gelation of Fmoc-F_5_-Phe-DAP. Hydrogels formulated with NaCl are acidic (pH ∼ 5) while hydrogels formulated with DMEM are neutral (pH 7).

**Figure 2:**
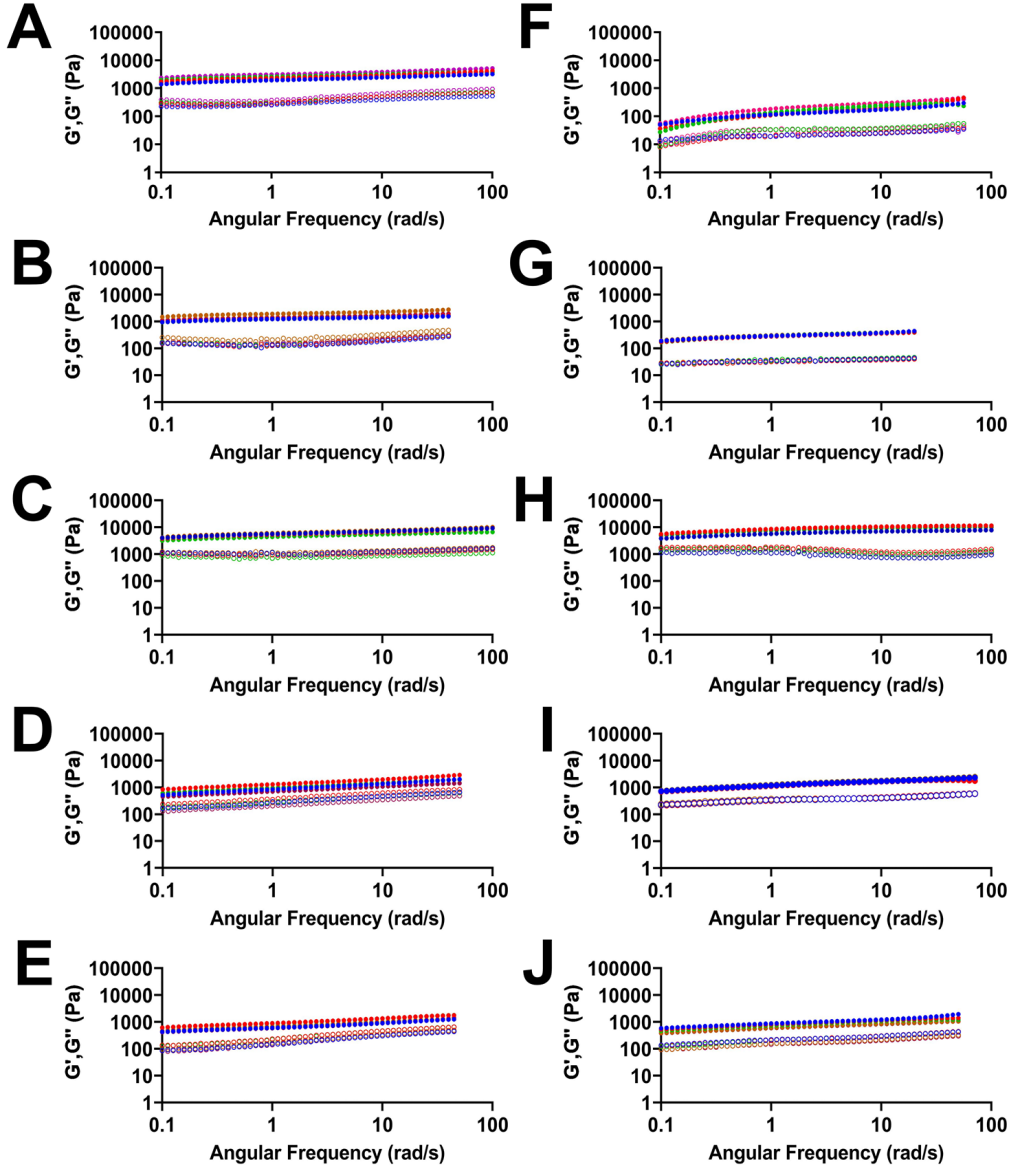
Oscillatory frequency sweep rheology of Fmoc-F_5_-Phe-DAP NaCl hydrogels loaded with model proteins. *G*′ and *G*″ values (Pa) are represented with closed and open circles, respectively. NaCl gels loaded with (A) no protein, (B) RNase A, (C) TI, (D), BSA, and (E) human IgG. DMEM gels loaded with (F) no protein, (G) RNase A, (H) TI, (I) BSA, and (J) human IgG. Five individual measurements of the same system are reported together in each graph.

The emergent viscoelastic properties of these hydrogels were characterized to determine the effects of protein encapsulation within the network. Oscillatory rheology was used to analyze the mechanical properties of our protein-loaded hydrogels (Figure 2). Specifically, frequency sweep experiments at strain values of 0.2% (which falls within the linear viscoelastic region for all gels as determined by strain sweep analysis; see Figures S11 and S12 in the Supporting Information for strain sweep data) were performed from 0.01 to 100 rad s^–1^. These experiments were used to determine the storage (*G*′) and loss (*G*″) moduli for each hydrogel. All hydrogels possessed storage moduli (*G*′) values that were approximately parallel and an order of magnitude greater than the loss moduli (*G*″) values, with *G*′ and *G*″ values parallel and separated by approximately an order of magnitude (Table 2).

**Table 2:** Average Storage (*G*′) and Loss (*G*″) Moduli of Protein-Loaded Hydrogels and Protein Charge^a^

	NaCl gels			DMEM gels		
protein	charge	*G*′ (Pa)	*G*″ (Pa)	charge	*G*′ (Pa)	*G*″ (Pa)
none		2782 ± 492	433 ± 77		196 ± 38	29 ± 6
RNase A	18.3^63^	1479 ± 318	195 ± 45	12.8^63^	300 ± 10	35 ± 2
TI	1.1^63^	6057 ± 800	1094 ± 179	–7.5^63^	7950 ± 1270	1258 ± 224
BSA	23.4^63^	1179 ± 269	366 ± 73	–9.9^63^	1426 ± 76	377 ± 4.5
human IgG^64^	54.2 ± 5.8	885 ± 176	264 ± 56	–1.1 ± 4.1^64^	842 ± 154	220 ± 38

a Error reported as standard deviation of the mean.

Moderate variability in hydrogel viscoelasticity was observed as a function of protein loading. The hydrogel formulation methods (NaCl versus DMEM) strongly impacted hydrogel viscoelasticity. Hydrogels without protein have average storage moduli of 2782 ± 492 and 196 ± 38 Pa for NaCl and DMEM gels, respectively. This large discrepancy in storage moduli (*G*′) is likely due to a reduction in the number of ions available in solution that screen positive charge in DMEM gels (∼33 mM total Na^+^, Ca^2+^, K^+^, and Mg^2+^ salts, with ∼22 mM NaCl), compared to the in NaCl gels (114 mM NaCl). BSA-loaded and human IgG-loaded NaCl gels were the weakest gels with similar viscoelasticity. The average storage moduli values were 1179 ± 269 and 885 ± 176 Pa for BSA and human IgG NaCl gels, respectively, with a tendency to increase with an increase in the angular frequency. RNase A-loaded NaCl gels had an average *G*′ of 1479 ± 318 Pa; however, this value remains constant with increasing angular frequency. Loading with TI provided the strongest hydrogels, with an average *G*′ of 6057 ± 800 Pa. The large discrepancy in *G*′ values between TI and our other model proteins may be a result of the propensity or TI to form decamers or clusters of decamers in the presence of the sodium chloride solution,^65^ leading to an overall increase in gel strength due to noncovalent cross-linking between TI oligomers and the hydrogel network.

Triggering gelation with DMEM led to protein-dependent variations in *G*′ values. Interestingly, loading DMEM gels with most of our model proteins resulted in a significant increase in hydrogel rigidity, compared to unloaded DMEM gels. BSA-loaded and human IgG-loaded gels demonstrated a small increase in *G*′ (1426 ± 76 Pa) and a small decrease (842 ± 154 Pa) compared to NaCl gels, respectively, while maintaining their tendency to increase with increasing angular frequency. TI-loaded gels remained the strongest, with a slight increase in *G*′ (7950 ± 1270 Pa). Gelation of RNase A-loaded hydrogels with DMEM resulted in a significant decrease in *G*′ (300 ± 10 Pa) compared to NaCl gels, resulting in significantly weaker hydrogels. The pI of RNase A (9.6) is significantly higher than for the other proteins used in this study (Table 1). Thus, the net charge of RNase A is estimated to be 18.3 at pH 5 and 12.8 at pH 7.^63^ This change further reduces the ions available to screen Fmoc-F_5_-Phe-DAP cations as well as the added cations of RNase A, thus reducing hydrogel viscoelasticity by decreasing the favorability of fibril/fibril interactions and protein/fibril interactions in the network.

**Figure 3:**
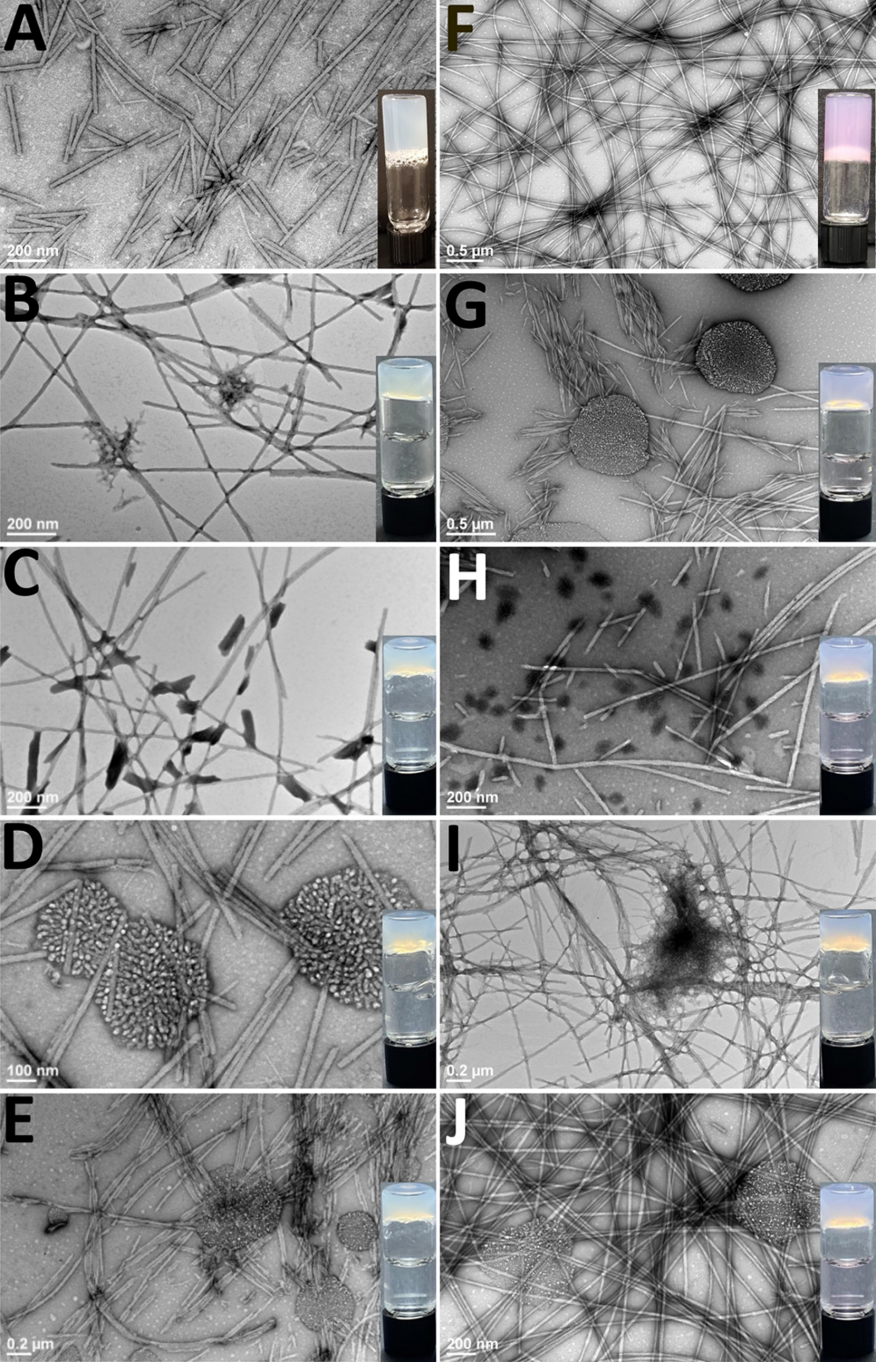
TEM of protein-loaded Fmoc-F_5_-Phe-DAP hydrogels. NaCl gels loaded with (A) no protein, (B) RNase A, (C) TI, (D), BSA, and (E) human IgG. DMEM gels loaded with (F) no protein, (G) RNase A, (H) TI, (I) BSA, and (J) human IgG.

Protein-loaded hydrogels were also characterized by transmission electron microscopy (TEM) (Figure 3 and Figure S11, Supporting Information). Transmission electron microscopy was performed on diluted samples of hydrogels to facilitate a clear assessment of the morphology of the self-assembled fibril that constitute the hydrogel network. The resulting TEM images (Figure 3 and S11, Supporting Information) thus provide insight into whether the presence of proteins perturbs the self-assembly of the gelators as evidenced by the fibril morphology. These images do not provide direct insight into the possible impact of proteins on the network formed by these fibrils that subsequently give rise to the emergent hydrogelation. We have previously reported that Fmoc-F_5_-Phe-DAP hydrogels form thin fibrils approximately 30.0 ± 4.3 nm in diameter when NaCl is used to increase the ionic strength in gel formulation (Figure 3A).^36,38^ Similar fibrils were observed in hydrogels formulated with DMEM (Figure 3F). Identical characteristic fibrils were observed in all protein-loaded hydrogels, regardless of NaCl or DMEM formulation. The protein-loaded gels also show protein aggregates that appear to be amorphous spherical micelles or inclusion body-like aggregates.^66−68^ RNase A-loaded NaCl gels contain smaller amorphous aggregates (Figure 3B), while larger, spherical aggregates are observed in the DMEM gels (Figure 3G). TI forms the smallest aggregates that appear as amorphous features in the TEM images and appear to be associated closely with the hydrogel fibrils (Figure 3C,H). In BSA-loaded NaCl gels, the protein appears as variably sized aggregates that look similar to protein inclusion bodies (Figure 3D). However, amorphous aggregates are predominately found in the BSA/DMEM gels (Figure 3I). Gels loaded with human IgG exhibit large, spherical inclusion body-like protein aggregates regardless of the gelation method (Figure 3E,J). TEM images of aqueous protein solutions (nonhydrogel) were also obtained (Figure S12, Supporting Information). These images show that the protein aggregate formation is not dependent on the hydrogel network but is also observed in NaCl and DMEM solutions.

These data show that protein encapsulation does influence the emergent viscoelasticity of supramolecular Fmoc-F_5_-Phe-DAP hydrogels. Interestingly, there doesn’t appear to be a clear correlation between protein size, isoelectric point, and emergent hydrogel viscoelasticity. Hydrogels formulated in NaCl solution (pH ∼ 5) increase in storage modulus in the order IgG < BSA ≈ RNase A < TI. This order is different in DMEM-formulated gels (pH ∼ 7), with storage modulus increasing in the order RNase A < IgG < BSA < TI. Regardless of the formulation method and protein loaded into the hydrogel, the TEM images indicate formation of fibrils approximately 20 nm in diameter for all cases (Figure S11 and Table S2), Supporting Information. Therefore, differences in hydrogel viscoelasticity cannot be attributed to perturbation of the fibril morphology. The efficiency of network cross-linking must therefore be altered by the proteins. TI and BSA have similar pI values (ca. 4.5–5), but the hydrogels containing these respective proteins have drastically different storage/loss moduli. The smaller positive charge (+1.1) of TI^63^ may increase fibril-fibril cross-linking by interacting more extensively with the network in its oligomeric forms resulting in increased mechanical rigidity of the gels, compared to more positively charged BSA (+23.4).^63^ Additionally, TI is the only protein that does not show inclusion of body-like aggregates in TEM images. Perhaps these types of aggregates (and the extent to which they form, which cannot be accurately estimated by TEM alone) may impact hydrogel rigidity. RNase A-containing gels show the most dramatic change in viscoelasticity as a function of formulation method, with NaCl gels having stronger storage modulus values than DMEM gels. As stated above, this may be due to the high pI of RNase A (9.6). At neutral pH (DMEM), RNase A is expected to be less positively charged, thus affecting interactions of RNase A with the hydrogel network and causing changes in the emergent viscoelasticity. In addition, the DMEM formulation has a lower net concentration of ions that assist in screening positive repulsive interactions, which will also reduce the noncovalent interactions that strengthen the hydrogel network.

It may also be that the amount of protein in the gels impacts the emergent viscoelasticity. Each of the hydrogels uniformly contain 1 mg mL^–1^ of the respective protein. However, the differing molecular weights of these proteins means that there is a significant difference in the molar concentration of the proteins in each gel. RNase A is present at 73 μM, TI at 50 μM, BSA at 15 μM, and IgG at 7 μM. Note that differences in protein solubility make standardization of protein amount by molarity impractical. For this reason, we calculated a normalized charge density introduced by the proteins in each system by multiplying the net charge of each protein at pH 5 for NaCl gels and at neutral pH for DMEM gels by the molar concentration of the proteins in each hydrogel (Table S3, Supporting Information). In the NaCl gels, the charge introduced by each protein is positive and decreases in the order of RNase A > IgG ≈ BSA > TI, with the normalized charge density ranging from 1343 for RNase A to 60 for TI, with BSA and IgG at intermediate charge densities of 353 and 379, respectively. For DMEM gels, the normalized charge density for RNase A is positive 942, while TI, BSA, and IgG are negative and decrease in the order TI (−380) > BSA (−150) > human IgG (−7.7) (Table S2).

This approach provides insight into the emergent viscoelasticity of the protein-loaded hydrogels. For the NaCl hydrogels, the viscoelasticity of the hydrogels does correlate with the normalized charge density, with the least positive system (TI) having the strongest viscoelasticity and the most positive system (IgG) having the weakest viscoelasticity. The NaCl gels with RNase A and BSA have similar charge densities and similar viscoelasticity that lies between that of the TI and IgG systems. These viscoelasticities are thus reflective of a lower density of positive charge from the protein cargo contributing to more minimal disruption of the hydrogel network, which has the effect of reducing noncovalent cross-linking within the network. For the DMEM hydrogels, the most negative system (TI) has the highest viscoelasticity, followed by BSA and IgG, indicative of reinforcement of the network by increased negative charge. The positive charge introduced by RNase in the DMEM hydrogels disrupts cross-linking in the network, thus reducing the viscoelasticity. The changes in viscoelasticity observed when comparing NaCl and DMEM gels are also explained by these normalized charge densities. For example, the modestly strengthened viscoelasticity of DMEM gels compared to NaCl gels with TI, BSA, and IgG correlates to a shift from positive protein charge in NaCl gels to negative charge in DMEM gels, which is expected to strengthen network interactions. This correlation is not perfect, however. The positive charge density of RNase A in DMEM gels is significantly lower than that observed in NaCl gels, which one would predict should strengthen the DMEM viscoelasticity. In fact, the viscoelasticity of the RNase A DMEM hydrogel is significantly weakened. This is likely due to the decreased ionic strength of DMEM (based on salt concentrations) compared to the NaCl system, which dominates the protein effect in this particular instance. In general, we can conclude that the viscoelasticity of Fmoc-F_5_-Phe-DAP hydrogels is strongly dependent on the ionic strength of the media and on the nature of the protein cargo. The charge introduced by a particular protein contributes to reinforcing or disruptive interactions within the hydrogel network, thus influencing the emergent viscoelasticity of the resulting networks.

### Controlled Release of Proteins from Supramolecular Fmoc-F_5_-Phe-DAP Hydrogels

**Figure 4:**
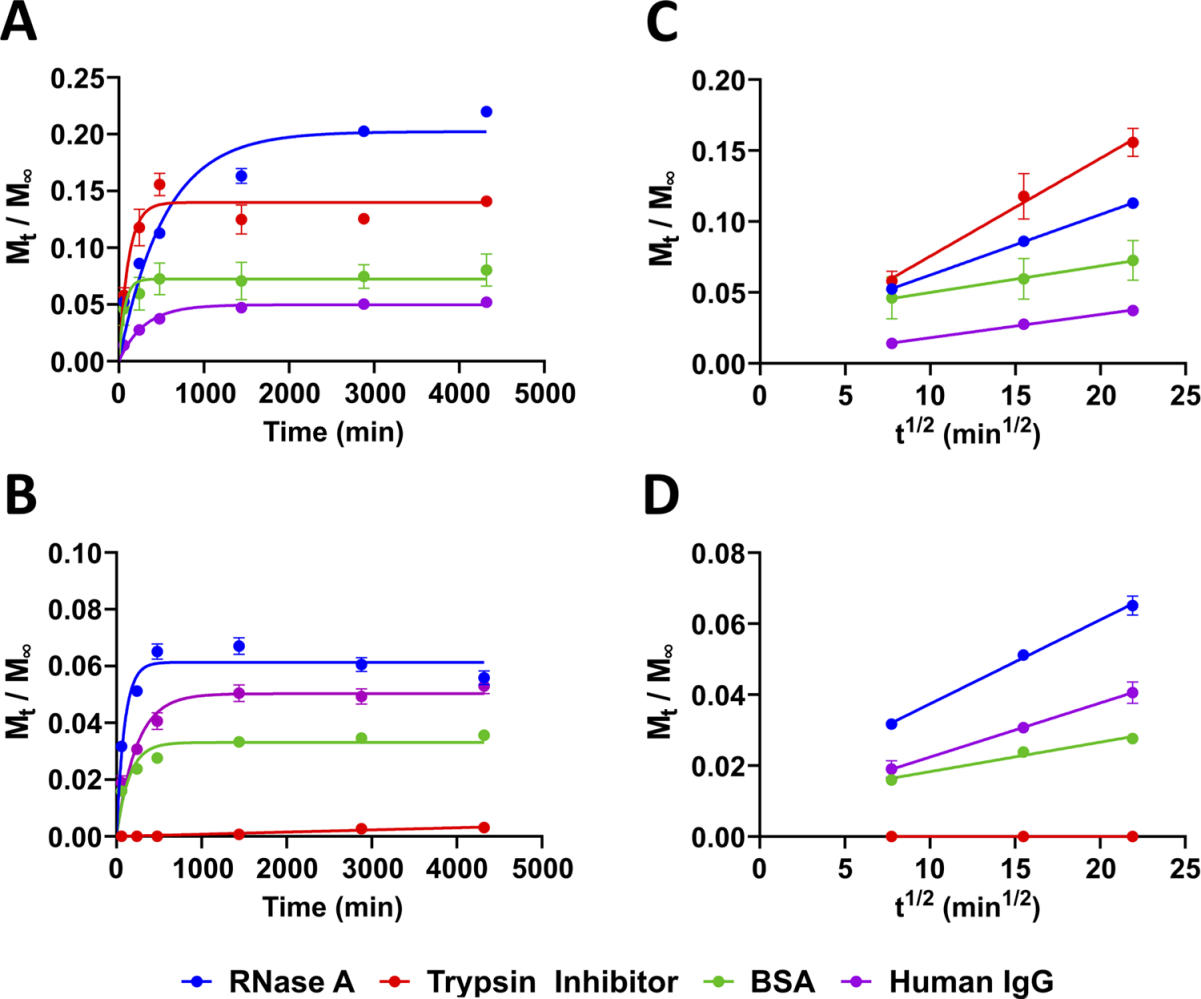
Release profiles of our model proteins in Fmoc-F_5_-Phe-DAP hydrogels formulated with (A) NaCl or (B) DMEM. The linear portion of the release curves, determined to be within the first 480 min, is plotted as *M_t_*/*M*_∞_ vs *t*^1/2^ for hydrogels formulated with (C) NaCl or (D) DMEM. The slope of the lines provides the diffusion coefficient, *D*, for each protein release profile.

Based on previous work demonstrating controlled release of small molecules from fluorinated Fmoc-F_5_-Phe-DAP hydrogels *in vitro* and *in vivo*, we next characterized the release profiles of each protein into solvent reservoirs layered over the various hydrogels.^37,38^ These studies were used to understand the release of proteins from the hydrogels over time. Hydrogels of Fmoc-F_5_-Phe-DAP (15 mM, 8.6 mg mL^–1^) loaded with model proteins (1 mg mL^–1^, 12.5% w/v) were formulated as described previously with either NaCl (114 mM, pH ∼ 5) or DMEM (33 mM, pH ∼ 7). After 12 h of equilibration, gels (0.5 mL) were layered with phosphate-buffered saline (PBS, pH 7.4,1 mL) and incubated at 37 °C. Aliquots of the layered PBS solution (100 μL) were removed at 1, 4, 8, 24, 48, and 72 h. After removing each aliquot, the buffer solution was immediately replenished by an equal volume of PBS (pH 7.4, 100 μL). The concentration of released protein was measured by quantification of protein concentration in the removed aliquots by correlation to an HPLC standard concentration curve (see Figures S7–S10 in the Supporting Information). The ratio of the amount of protein released after *t* hours to the total amount loaded into the hydrogel (*M*_*t*_/*M*_∞_) was plotted against time (*t*, min) (Table 3 and Figure 4A,B). Additionally, the diffusion coefficient, *D* (m^2^ min^–1^), was determined by plotting *M*_*t*_/*M*_∞_ against *t*^1/2^ (min^1/2^) for the initial linear release data observed in the first 480 min (Table 3 and Figure 4C,D) using the non-steady-state diffusion model outlined in eq 3 (see the Materials and Methods section). The diffusion coefficient, *D*, was determined from the slope of these data using eq 2.

**Table 3:** Average Protein Released after 72 h (%) and Extrapolated Diffusion Coefficients^a^

protein	total protein released (%), NaCl gels	total protein released (%), DMEM gels	diffusion coefficient (*D*, m^2^ min^–1^) in NaCl gels	diffusion coefficient (*D*, m^2^ min^–1^) in DMEM gels
RNase A	22.0 ± 0.5	5.6 ± 0.2	1.99 × 10^–12^ ± 1.1 × 10^–13^	2.26 × 10^–13^ ± 4.2 × 10^–14^
TI	14.1 ± 0.5	0.31 ± 0.01	7.25 × 10^–12^ ± 1.1 × 10^–13^	
BSA	8.0 ± 1.4	3.5 ± 0.1	1.69 × 10^–13^ ± 5.5 × 10^–14^	4.83 × 10^–15^ ± 4.2 × 10^–16^
human IgG	5.2 ± 0.1	5.3 ± 0.3	2.94 × 10^–14^ ± 2.3 × 10^–15^	2.21 × 10^–14^ ± 1.6 × 10^–15^

a Error is represented as standard deviation of the mean.

The total protein released was found to be variable as a function of both protein and gel formulation conditions. Total protein release from NaCl Fmoc-F_5_-Phe-DAP hydrogels over 72 h increases in the following order: IgG < BSA < TI < RNase A (Figure 4A). The large release of RNase A (22.0 ± 0.5% of total loaded protein) was expected since this is the smallest protein and is cationic at acidic pH. The cationic hydrogel was expected to retain RNase A to a lesser extent due to repulsive charge effects, as has been observed for small-molecule cargo in these supramolecular hydrogels.^38^ Even though human IgG is cationic at acidic pH, this protein was released to the smallest extent (5.2 ± 0.01%) with the slowest diffusion constant (2.94 × 10^–14^ m^2^ min^–1^). We hypothesize that the extreme size discrepancy between RNase A (13.7 kDa) and human IgG (150 kDa) accounts for the large difference in their release from the network. At the acidic pH of these hydrogels, TI (pI 4.5) and BSA (pI 4.7) are roughly neutral, which we hypothesized would result in release profiles primarily dependent on molecular weight. TI was released to a modest extent (14.1 ± 0.5%) with a rate of diffusion (7.25 × 10^–12^ m^2^ min^–1^) similar to RNase A, while release of BSA was only slightly increased over human IgG (8.0 ± 1.5%; 1.69 × 10^–13^ m^2^ min^–1^).

The mesh size of the hydrogel network is also likely to influence the release rates of proteins from these hydrogels, with smaller proteins released at more rapid rates. To gain insight into the relationship between hydrogel mesh size and protein release profiles we calculated the approximate mesh size of each hydrogel from the average storage moduli (*G*′) using eq 1 as previously reported by Shibayama^50^ and Webber (see the Materials and Methods section and Table S4 in the Supporting Information for calculated mesh size values).^49^ For NaCl hydrogels, the mesh size slightly increases in the following order: TI (9.0 nm) < RNase A (14.3 nm) < BSA (15.4 nm) < human IgG (17.0 nm). Although this trend is inversely proportional to *G*′ (Table S4), there is no clear correlation between the release profiles and rate of diffusion with the calculated mesh sizes. The apparent differences in mesh size in the hydrogels studied herein are relatively subtle, with a difference of less than 2-fold between the smallest and largest mesh sizes, compared to the differences in protein size and charge. Therefore, this data indicates that the protein release characteristics of the NaCl hydrogels is primarily dependent on the molecular weight and charge of the protein cargo.

Protein release profiles from DMEM-formulated hydrogels were found to deviate from release profiles observed in NaCl hydrogels. Total protein released from DMEM hydrogels increased in the following order: TI < BSA < Human IgG < RNase A (Figure 4B and Table S4). Interestingly, the total amount of protein released from DMEM gels was significantly reduced for all proteins with the exception of IgG, which was released in similar quantities from both DMEM and NaCl gels. For example, total RNase A released (5.6 ± 0.2%) and its rate of diffusion (2.26 × 10^–13^ m^2^ min^–1^) from the DMEM gels was significantly reduced compared to RNase A-loaded NaCl gels (22% and 1.99 × 10^–12^, respectively). We also calculated the approximate mesh size of the protein-loaded DMEM gels, which showed an increase in mesh size in the following order: TI (8.2 nm) < BSA (14.5 nm) < IgG (17.3 nm) < RNase A (24.4 nm) (see Table S4). There was a significant increase in mesh size for RNase A-loaded DMEM gels (24.4 nm) compared to the RNase A-loaded NaCl gels (14.3 nm). Although one would expect to observe increased RNase A release with an increased mesh size in DMEM gels, RNase A release amount and rates are actually lower for the DMEM hydrogels. The reduction of positive charge in RNase A at neutral pH (12.8) compared to acidic pH (18.3) likely accounts for these differences. The total release of human IgG (5.3 ± 0.3%), its rate of diffusion (2.21 × 10^–14^ m^2^ min^–1^), and the mesh size of these hydrogels (17.3 nm) remained consistent, regardless of gelation method, even though IgG is reported to be cationic at acidic pH (54.2 ± 5.8) and roughly neutral at physiological pH (−1.1 ± 4.1).^64^ Therefore, we conclude that protein release becomes primarily dependent on molecular weight rather than electrostatic interactions above a specific threshold. As expected, the increased pH of these gels had the strongest impact on TI and BSA release, the proteins with the lowest pI values. The total release (3.6 ± 0.5%) and rate of diffusion (4.83 × 10^–15^ m^2^ min^–1^) of BSA were both significantly reduced compared to NaCl hydrogels, while the mesh size of the materials remained unchanged (14.5 nm). The strongest impact was observed for TI, with only 0.31 ± 0.01% of loaded TI released after 72 h; the diffusion constant is negligible, indicating that most TI is retained in the network under these gelation conditions even though the hydrogel mesh size remained relatively unchanged (8.2 nm) compared to the NaCl gels. Decreased release of TI and BSA from DMEM gels compared to NaCl gels is logical based on the effect of pH on protein charge in these systems. At neutral pH (DMEM gels), these proteins become negatively charged, resulting in stronger attractive interactions between the proteins and the cationic hydrogel network.

### Validation of Retention of Function of Released Proteins

Thus far, we have demonstrated that proteins of varying size and charge can be released from our hydrogels and that their primary structure remains unchanged. To determine if protein functionality is unaltered, we employed a variety of assays to test native functions and further validate the native structures of our proteins. First, we utilized polyacrylamide gel electrophoresis (PAGE) to validate that released proteins retain their native primary structures and that the protein chains are not degraded (Figure S13). RNase A, TI, and BSA are composed of a single polypeptide chain, while human IgG consists of multiple polypeptide chains connected via disulfide linkages. SDS-PAGE confirmed single protein bands for RNase A, TI, and BSA, matching their native counterparts loaded as controls (Figure S13A–C). Under denaturing conditions, human IgG shows two bands at 50 and 25 kDa; while under nondenaturing conditions, human IgG remains a single band at the top of the gel (Figure S13D,E). Therefore, we can conclude that proteins released from our hydrogels maintain their primary structure.

### Enzymatic Activity of RNase A

**Figure 5:**
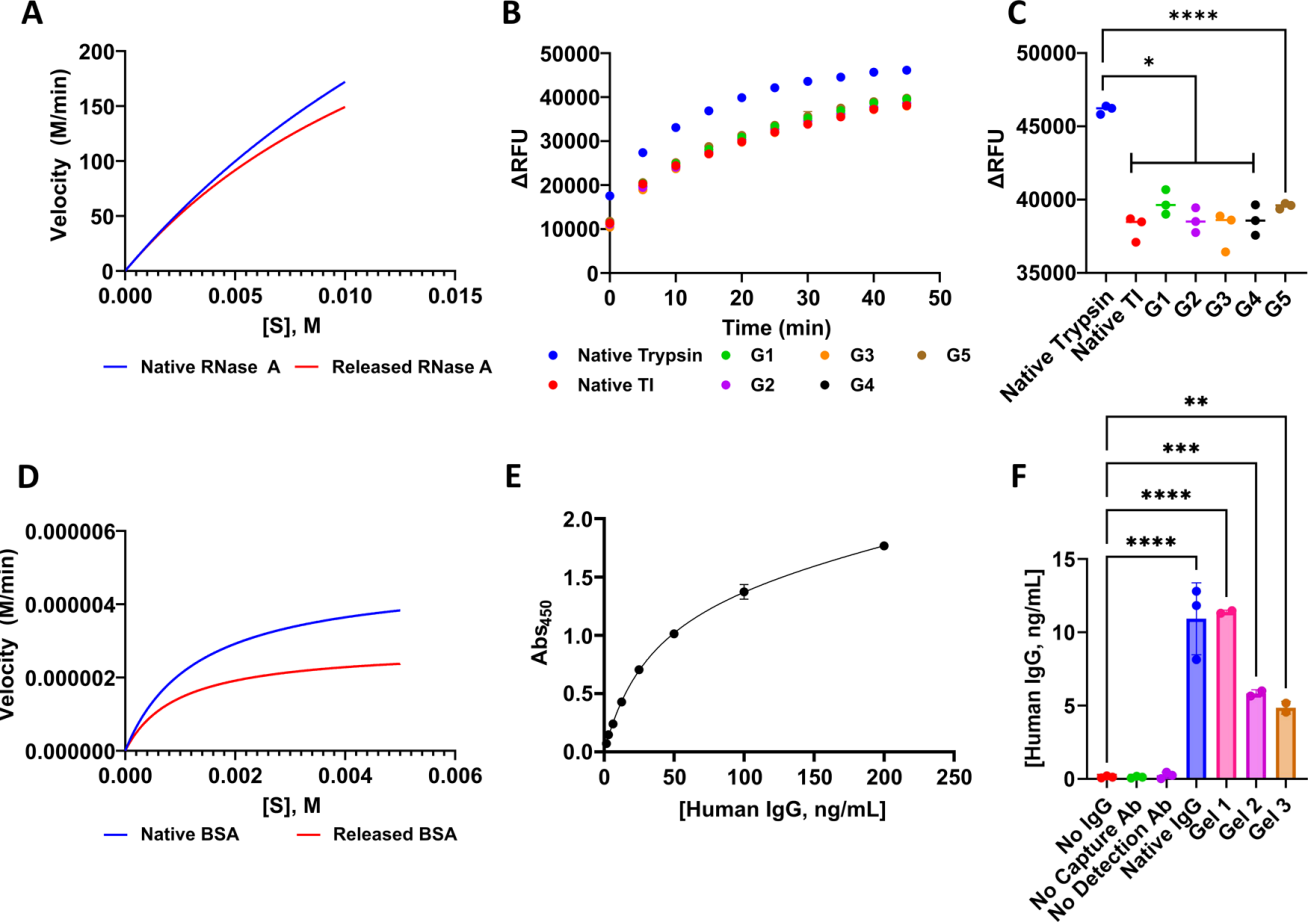
(A) Michaelis–Menten plot of native and released RNase A. (B) Change in relative fluorescence units (ΔRFU) of FITC-casein substrate subject to proteolysis by native trypsin in the presence or absence of TI over 45 min. G1–G5 denote supernatant taken from five different gels loaded with TI. (C) ΔRFU of FITC-casein substrate after 45 min of native trypsin, native trypsin inhibitor, and supernatant from five different gels loaded with TI (G1–G5). (*n* = 3, *p ≤ 0.05, ****p ≤ 0.0001). (D) Michaelis–Menten plot of native BSA and released BSA. (E) Standard curve generated from sandwich ELISA using Human IgG standard. (F) Human IgG concentrations (ng mL^–1^) of controls and supernatant from three different gels loaded with Human IgG interpolated from the standard curve in (E). (*n* = 3, **p ≤ 0.01, ***p ≤ 0.001, ****p < 0.0001).

We then confirmed that RNase A retains its native ribonucleolytic function by validating the enzymatic activity of released RNase A. Ribonucleases catalyze the degradation of RNA via endonucleolytic cleavage of 3′-phosphomononucleotides via 2′,3′-cyclic intermediates.^69^ To confirm similar Michaelis–Menten kinetics of released RNase A with native RNase A, we measured comparative hydrolysis of cytidine 2′,3′-cyclic monophosphate (cCMP) by monitoring the increase in absorption at 295 nm for the released and native protein.^55^ We measured Abs_295_ every 0.4 s for 10 min of native released RNase A (1 μM) combined with various concentrations of cCMP (1, 2, 3, 4, 5, or 10 mM), and plotted the resulting Michaelis–Menten plots (Figure 5A, see also Figure S14 in the Supporting Information) with *V*_max_ (M min^–1^), *K*_m_ (M), *k*_cat_ (s^–1^), and catalytic efficiency (*k*_cat_/*K*_m_, M^–1^ s^–1^) values presented in Table 4. The measured catalytic efficiencies of native (3.95 × 10^5^ M^–1^ s^–1^) and released (3.98 × 10^5^ M^–1^ s^–1^) are virtually identical, confirming that protein released from supramolecular Fmoc-F_5_-Phe-DAP hydrogels retains approximately 100% of its enzymatic capacity.

**Table 4:** Enzymatic Kinetics Data Calculated from Michaelis–Menten Plots of Native and Released RNase A and BSA

protein	*V*_max_(M min^–1^)	*K*_m_ (M)	*k*_cat_ (s^–1^)	*k*_cat_/*K*_m_ (M^–1^ s^–1^)
native RNase A	232	3.07 × 10^–3^	1.05 × 10^4^	3.95 × 10^5^
released RNase A	94.7	6.04 × 10^–3^	6.69 × 10^3^	3.98 × 10^5^
native BSA	4.84 × 10^–6^	1.31 × 10^–3^	8.06 × 10^–8^	6.15 × 10^–5^
released BSA	2.83 × 10^–6^	9.58 × 10^–4^	4.72 × 10^–8^	4.92 × 10^–5^

### Evaluation of Trypsin Activity in the Presence of Released Trypsin Inhibitor

TI is a serine protease inhibitor that acts to inhibit trypsin proteins by causing hydrolysis of peptide bonds in the active site to generate a stable enzyme-inhibitor complex.^70^ To verify that TI released from our hydrogels maintains its native function, we employed a FRET-based assay to measure the proteolytic activity of trypsin with a FITC-labeled substrate using a Thermo Scientific Pierce Fluorescent Protease Activity Kit.^59,60^ Fluorescence properties of the FITC-casein substrate change dramatically upon proteolytic degradation by native trypsin. We incubated the FITC-casein substrate (10 μg mL^–1^) with native trypsin (500 ng mL^–1^) or native trypsin pre-incubated with native TI (500 ng mL^–1^) or supernatant from five gels loaded with TI and measured fluorescence intensity every 5 min for 45 min. We plotted the change in fluorescence intensity compared to FITC-casein alone (ΔRFU) as a function of time (min) (Figure 5B) and the change in fluorescence intensity (ΔRFU) of each sample after 45 min (Figure 5C). Incubation of native trypsin with released TI resulted in approximately 17% reduction in ΔRFU, which is not significantly different than native TI, which reduced ΔRFU by 16%. Our results demonstrate that TI released from our hydrogels maintains the ability to effectively inhibit native trypsin activity.

### Enzymatic Activity of BSA

BSA has been reported to demonstrate esterase activity.^71−73^ To measure Michaelis–Menten kinetics of our released BSA, we optimized an assay^56^ that monitors the hydrolysis of *p*-nitrophenyl acetate to *p*-nitrophenol by monitoring the increase in absorption at 401 nm every 0.4 s for 10 min of native or released BSA (10 μM) combined with serial dilutions of *p*-nitrophenyl acetate and plotted the resulting Michaelis–Menten plots (Figure 5D, see also Figure S15 in the Supporting Information) with *V*_max_ (M min^–1^), *K*_m_ (M), *k*_cat_ (s^–1^), and catalytic efficiency (*k*_cat_/*K*_m_, M^–1^ s^–1^) values outlined in Table 4. Released BSA was calculated to have a lower *V*_max_ (2.83 × 10^–6^ M min^–1^) than native BSA (4.84 × 10^–6^ M min^–1^). However, calculated catalytic efficiencies of native BSA (6.15 × 10^–5^ M^–1^ s^–1^) and released BSA (4.92 × 10^–5^ M^–1^ s^–1^) demonstrate that protein released from our hydrogels retain approximately 80% of its enzymatic capacity.

### Verification of Secondary Structure of Release Human IgG Via Sandwich ELISA

Although human IgG demonstrates no enzymatic or inhibitory activity, we utilized an Invitrogen IgG (Total) Human ELISA kit to verify the structural integrity of released human IgG.^74,75^ This kit utilizes a sandwich ELISA platform, in which an anti-human IgG capture monoclonal antibody coated on the ELISA plate binds an epitope of human IgG, while another horseradish peroxidase (HRP)-conjugated anti-human IgG detection monoclonal antibody binds a secondary epitope of the captured human IgG. Therefore, this platform would only give a positive signal for captured human IgG that retains its secondary structure. In addition to the introduction of our released human IgG and a native human IgG positive control, we generated a standard curve (Figure 5E) from the serial dilution of a human IgG standard provided with the kit. The results of the assay are outlined in Figure 5F and the concentration of protein from three different hydrogels obtained at different timepoints was extrapolated using the standard curve. This data demonstrates that human IgG released from our hydrogels retains its secondary structure and ability to bind at various epitopes.

## Conclusions

In conclusion, we have demonstrated that LMW Fmoc-F_5_-Phe-DAP supramolecular hydrogels possess the requisite emergent properties for the sustained release of functional proteins. The hydrogels facilitate the release of proteins of varying size and charge, and release rates can be controlled by modifying the pH and ionic strength of the hydrogel network. Significantly, the proteins maintain native function within the hydrogel network and upon release from the network. These supramolecular hydrogels have significant advantages over polymer and supramolecular peptide hydrogels. Fmoc-F_5_-Phe-DAP is significantly less expensive to synthesize and purify relative to comparative self-assembled peptides used in hydrogel formulations. In addition, we have previously shown that Fmoc-Phe-derived hydrogels are deliverable by noninvasive injection and are well tolerated *in vivo*.^37^ The release of proteins that retain function from these LMW hydrogels provides validation that these biomaterials are viable vectors for the delivery of protein therapeutics, which are a prominent class of emerging pharmacologic agents. These hydrogels thus have great promise as next-generation biomaterials for the localized and sustained release of therapeutic protein biomacromolecules.

## Supporting Information Available

TEM images of model proteins in solution and of Fmoc-F_5_-Phe-DAP hydrogels, fibril width measurements, calculated charge density values, calculated mesh size values, analytical HPLC conditions, analytical HPLC traces of proteins, protein concentration curves, strain sweep oscillatory rheology data, SDS-PAGE analysis of release proteins, and supporting data for functional protein assays (PDF)
