# Supplementary material for: Supramolecular Phenylalanine-Derived Hydrogels for the Sustained Release of Functional Proteins
Source: ACS Biomater Sci Eng. 2023 Jan 24;9(2):784–96. doi: 10.1021/acsbiomaterials.2c01299 (PMC9930093; doi:10.1021/acsbiomaterials.2c01299)
Supplement: Supplementary file 1 — ab2c01299_si_001.pdf [file ab2c01299_si_001.pdf]

Supramolecular Phenylalanine-Derived Hydrogels for the Sustained Release of Functional Proteins

*Melissa L. Jagrosse,[a] Pamela Agredo,[a] Brittany L. Abraham,[a] Ethan S. Toriki,[a] and Bradley L. Nilsson[a,b]**

[a]Department of Chemistry, University of Rochester, Rochester, NY 14627, USA.

[b]Materials Science Program, University of Rochester, Rochester, NY 14627, USA.

E-mail: bradley.nilsson@rochester.edu

Tel. +1 585 276-3053

SUPPORTING INFORMATION

CONTENTS

Figure S1. Strain sweep data collected via oscillatory rheology for NaCl gels. G′ and G″values (Pa) are represented by closed and open circles, respectively. **(A)** Unloaded hydrogel. **(B)** RNase A-loaded hydrogel. **(C)** TI-loaded hydrogel. **(D)** BSA-loaded hydrogel. **(E)** Human IgG-loaded hydrogel. 3

Figure S2. Strain sweep data collected via oscillatory rheology for DMEM gels. G′ and G″ values (Pa) are represented by closed and open circles, respectively. **(A)** Unloaded hydrogel. **(B)** RNase A-loaded hydrogel. **(C)** TI-loaded hydrogel. **(D)** BSA-loaded hydrogel. **(E)** Human IgG-loaded hydrogel. 4

Table S1. Analytical HPLC analysis conditions. 5

Figure S3. Analytical HPLC trace of RNase A at 215 nm. 5

Figure S4. Analytical HPLC trace of TI at 215 nm. 6

Figure S5. Analytical HPLC trace of BSA at 215 nm. 6

Figure S6. Analytical HPLC trace of Human IgG at 215 nm. 7

Figure S7. Concentration curve of RNase A 7

Figure S8. Concentration curve of TI. 8

Figure S9. Concentration curve of BSA. 8

Figure S10. Concentration curve of Human IgG. 9

Figure S11. TEM images of Fmoc-F_5-Phe-DAP hydrogels. NaCl gels loaded with **(A)** RNase A, **(B)** TI, **(C)** BSA, **(D)**, human IgG. DMEM gels loaded with **(E)** RNase A, **(F)** TI, **(G)** BSA, and **(H)** human IgG. 10

Figure S12. TEM images of model proteins. NaCl solutions containing (A) RNase A, (B) TI, (C) BSA, and (D) Human IgG. DMEM solutions containing (E) RNase A, (F) TI, (G) BSA, and (H) Human IgG. 10

Table S2. Fibril width measurements for all systems with and without proteins loaded 11

Table S3. The normalized charge density of each system was calculated by multiplying the net charge by the molar concentration of each protein. 11

Table S4. Calculated mesh size for each hydrogel. The molecular weight and charge of each protein are also shown for comparison purposes with the % protein released and the diffusion coefficient. 12

Figure S13. SDS-PAGE of native and released proteins. **(A)** RNase A. Lanes contain (1) Bio-Rad Precision Plus Dual Color Protein Standard, (2-3) native RNase A, and (4-5) released RNase A. **(B)** Western Blot of TI. Lanes contain (1) Bio-Rad Precision Plus Dual Color Protein Standard, (2) native TI, and (3-6) released TI. **(C)** Western blot of BSA. Lanes contain (1) Bio-Rad Precision Plus Dual Color Protein Standard, (2) native BSA, and (3-5) released BSA. **(D)** SDS-PAGE of Human IgG in the presence of β-mercaptoethanol. Lanes contain (1) Bio-Rad Precision Plus Dual Color Protein Standard, (2) native Human IgG, and (3-5) released Human IgG. Image was cropped to remove unrelated samples and place human IgG lanes close to reference ladder. **(E)** SDS-PAGE of Human IgG in the absence of β-mercaptoethanol. Lanes contain (1) Bio-Rad Precision Plus Dual Color Protein Standard, (2) native Human IgG, and (3-5) released Human IgG. Image was cropped to remove unrelated samples and place human IgG lanes close to reference ladder. 13

Figure S14. Change in absorbance (295 nm) vs time (s) of **(A)** native and **(B)** released RNase A incubated with varying concentrations of cCMP. 14

Figure S15. Change in absorbance (401 nm) vs time (s) of **(A)** native and **(B)** released BSA incubated with varying concentrations of *p*-nitrophenyl acetate. 14

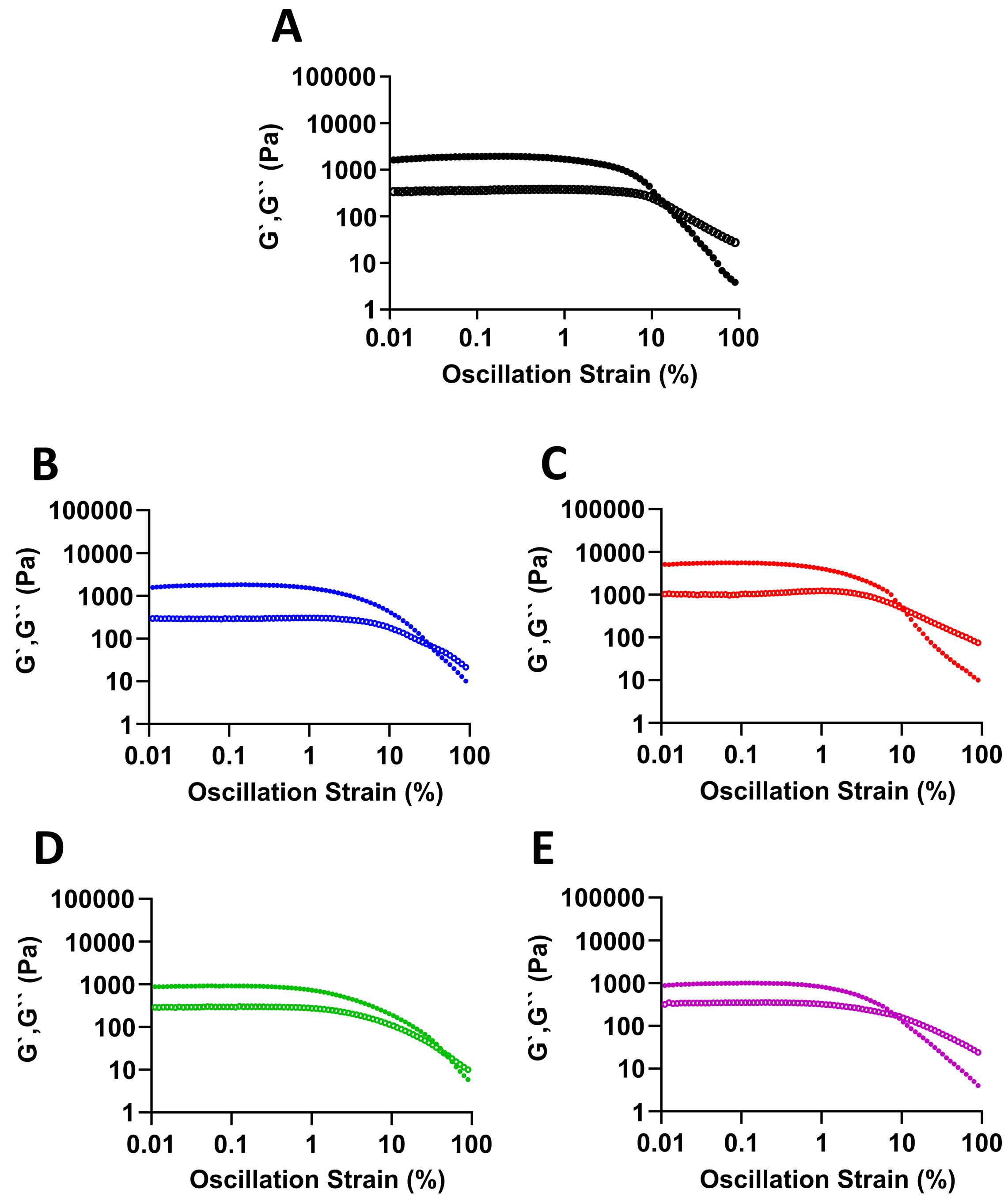

Figure S1. Strain sweep data collected via oscillatory rheology for NaCl gels. G′ and G″values (Pa) are represented by closed and open circles, respectively. **(A)** Unloaded hydrogel. **(B)** RNase A-loaded hydrogel. **(C)** TI-loaded hydrogel. **(D)** BSA-loaded hydrogel. **(E)** Human IgG-loaded hydrogel.

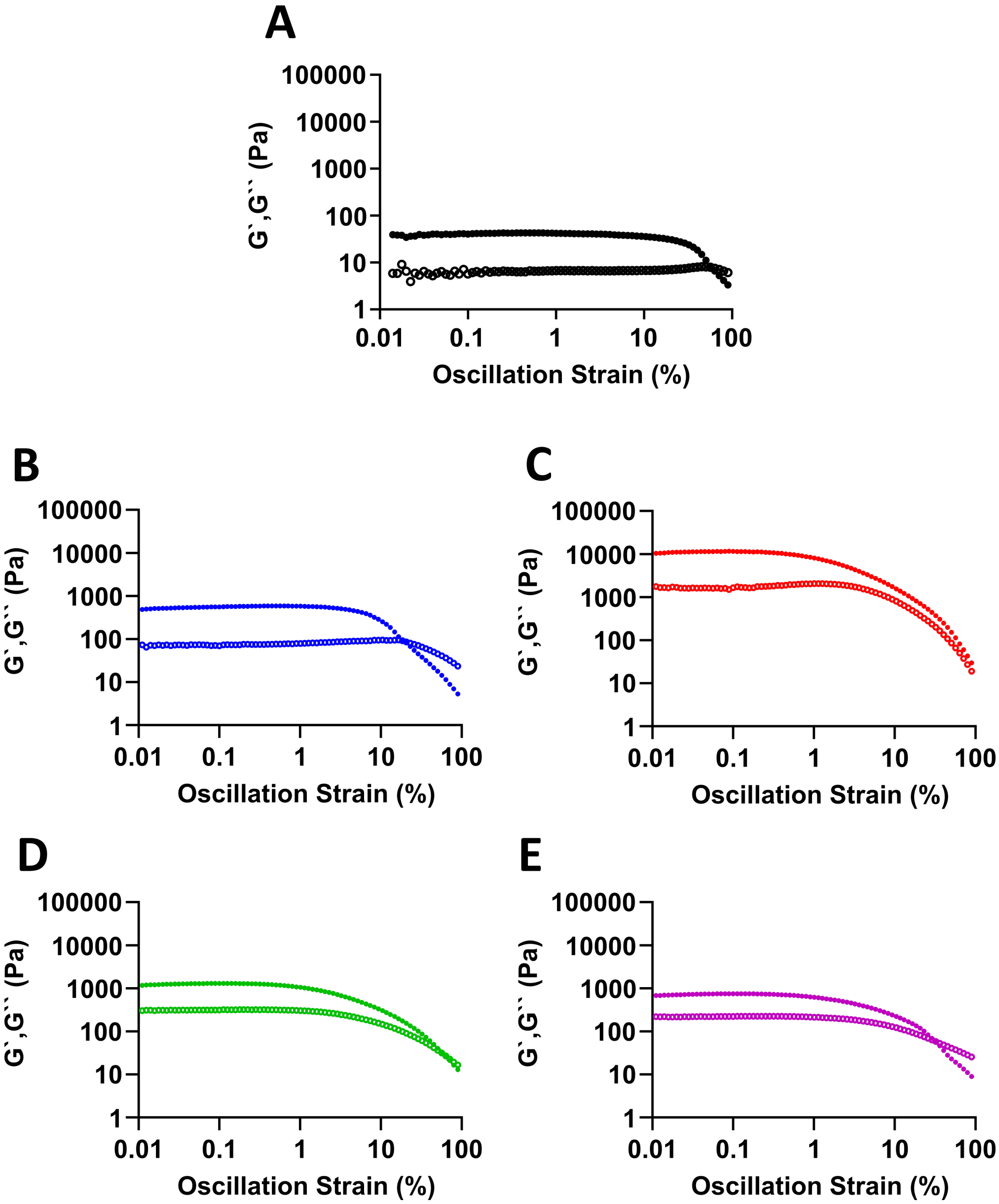

Figure S2. Strain sweep data collected via oscillatory rheology for DMEM gels. G′ and G″ values (Pa) are represented by closed and open circles, respectively. **(A)** Unloaded hydrogel. **(B)** RNase A-loaded hydrogel. **(C)** TI-loaded hydrogel. **(D)** BSA-loaded hydrogel. **(E)** Human IgG-loaded hydrogel.

Table S1. Analytical HPLC analysis conditions.

**Protein**	**R_t** (min)	**Gradient (solution** A**: water/0.5% TFA; solution** B**: acetonitrile/0.5% TFA)**	**Temperature (°C)**
RNase A	11.528	Isocratic 5% **B** 5 min, 5-95% **B** over 10min, 95% **B** 5 min	40
Trypsin Inhibitor	12.726	Isocratic 5% **B** 5 min, 5-95% **B** over 10min, 95% **B** 5 min	45
BSA	12.762	Isocratic 5% **B** 5 min, 5-95% **B** over 10min, 95% **B** 5 min	45
Human IgG	12.644	Isocratic 5% **B** 5 min, 5-95% **B** over 10min, 95% **B** 5 min	50

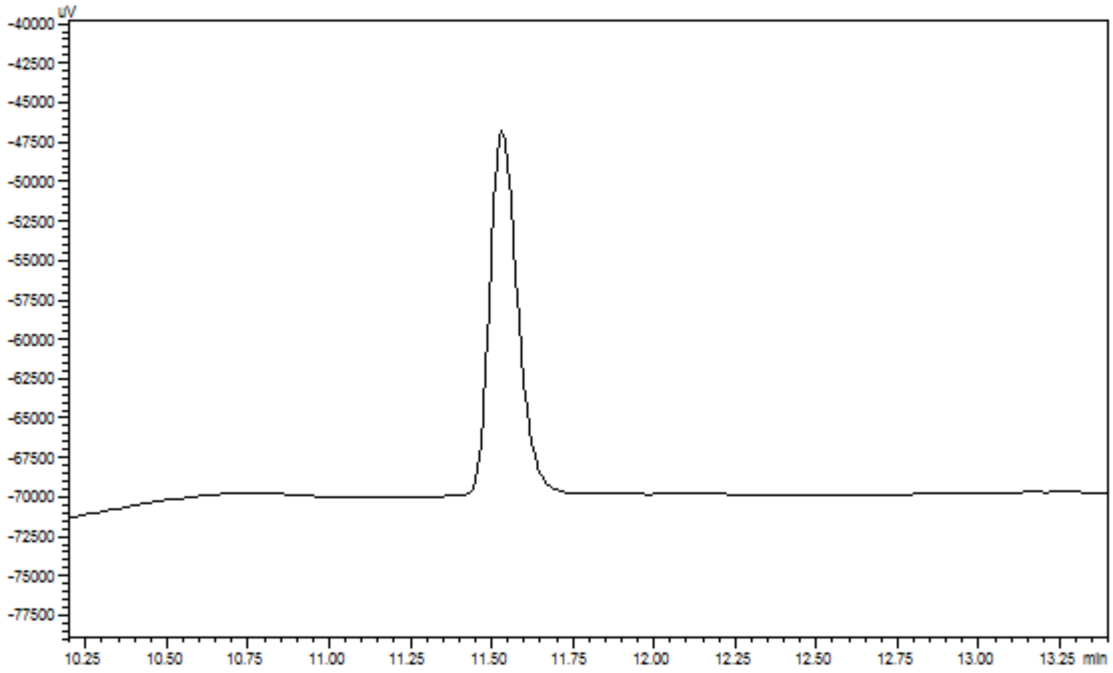

Figure S3. Analytical HPLC trace of RNase A at 215 nm.

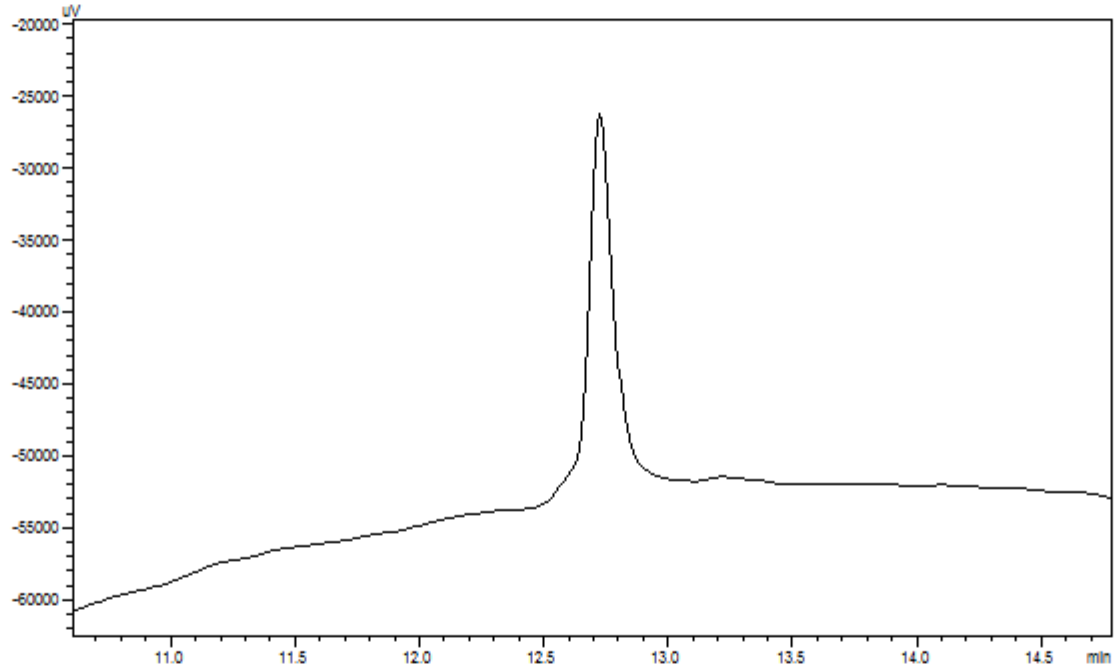

Figure S4. Analytical HPLC trace of TI at 215 nm.

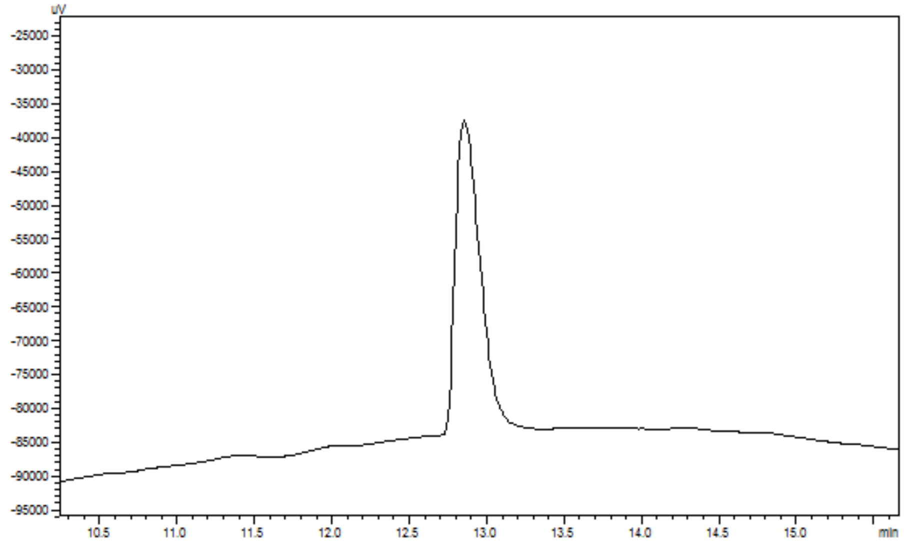

Figure S5. Analytical HPLC trace of BSA at 215 nm.

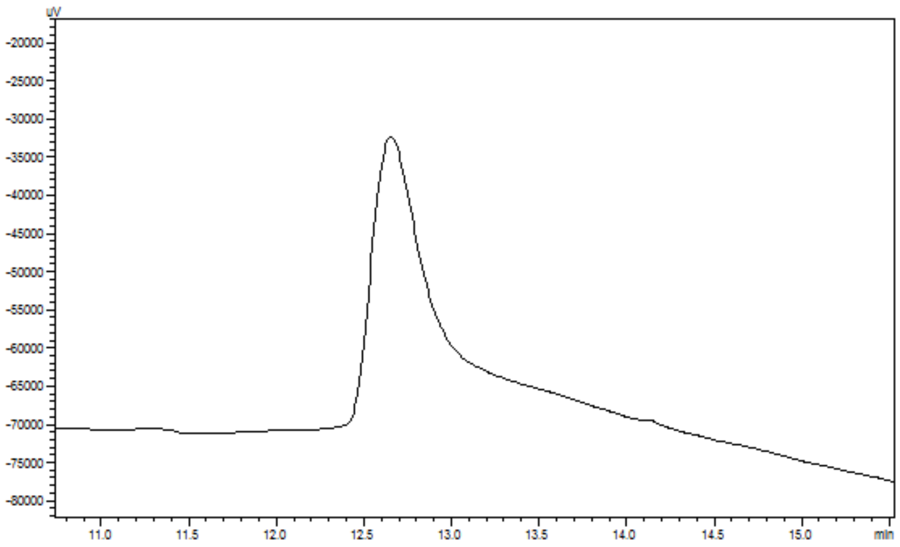

Figure S6. Analytical HPLC trace of Human IgG at 215 nm.

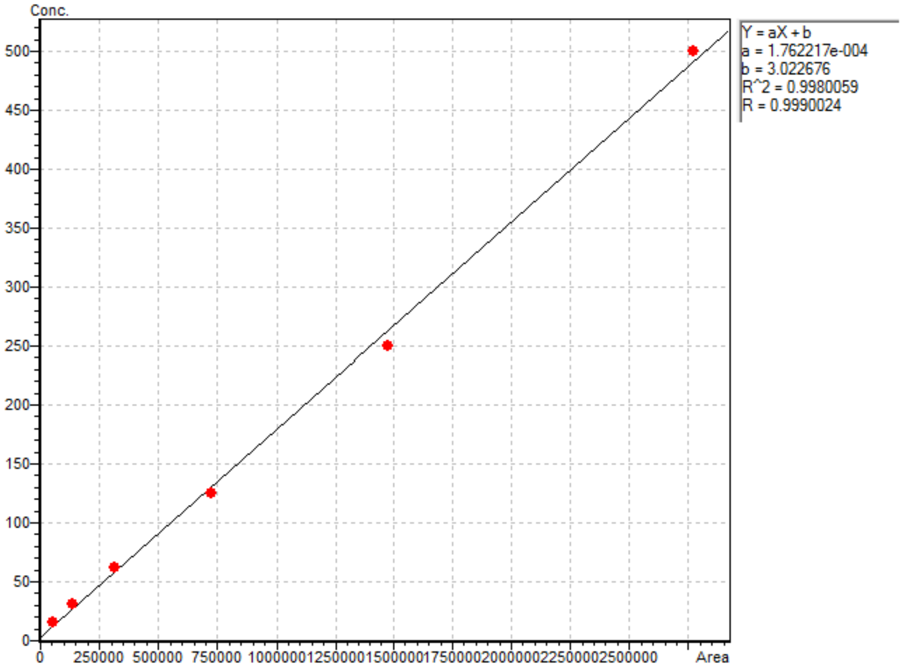

Figure S7. Concentration curve of RNase A

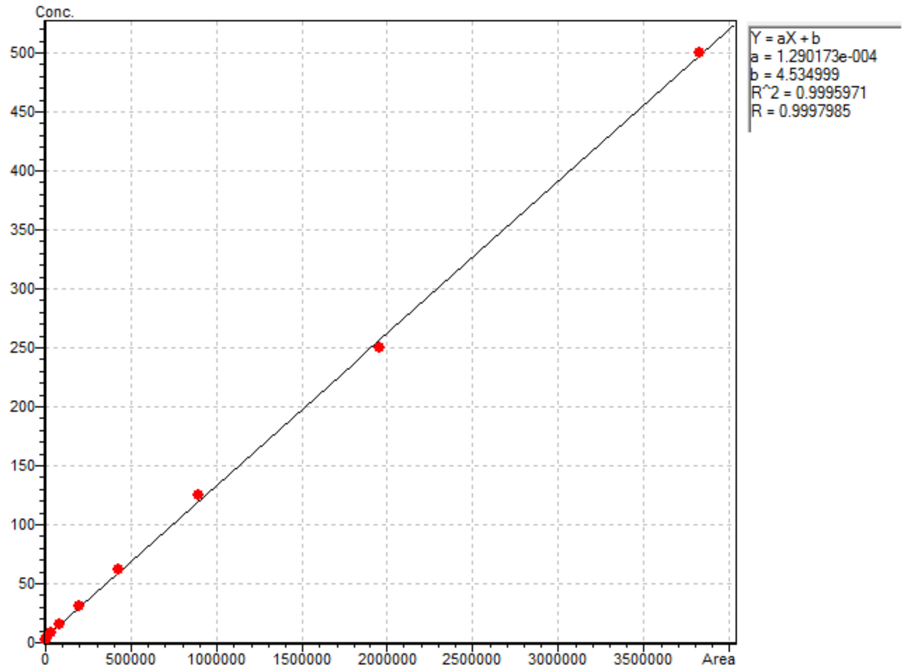

Figure S8. Concentration curve of TI.

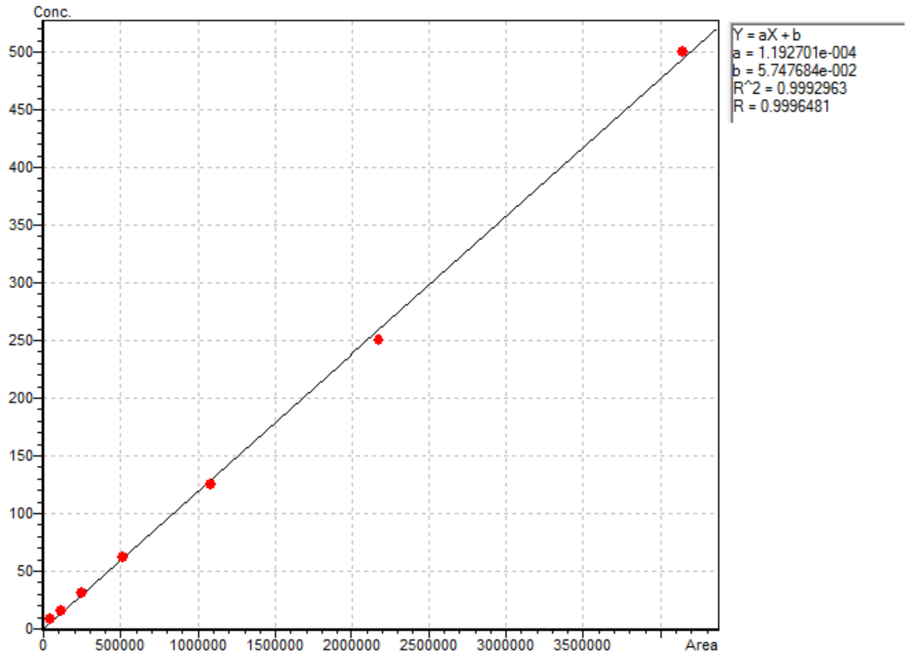

Figure S9. Concentration curve of BSA.

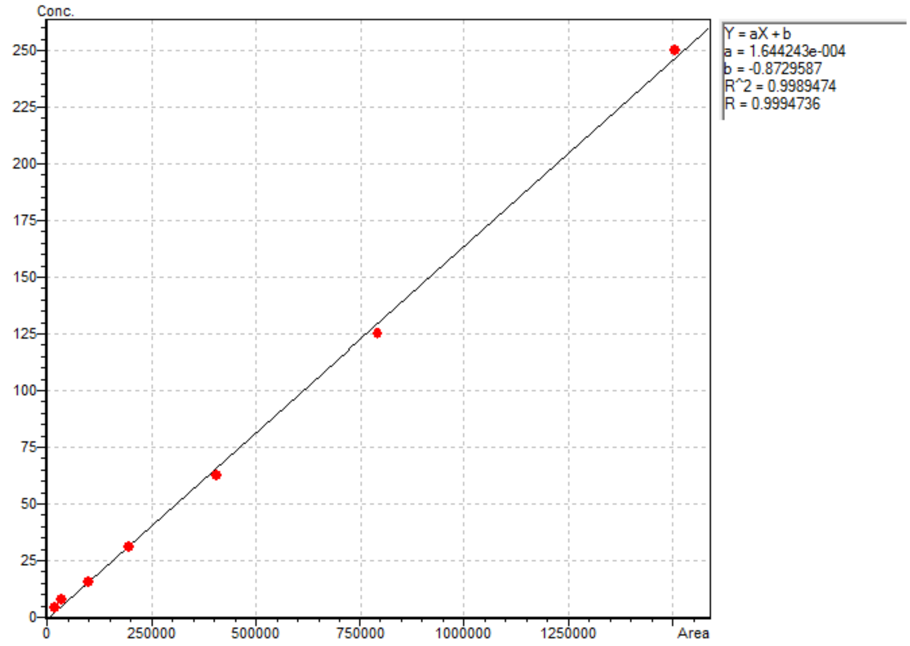

Figure S10. Concentration curve of Human IgG.

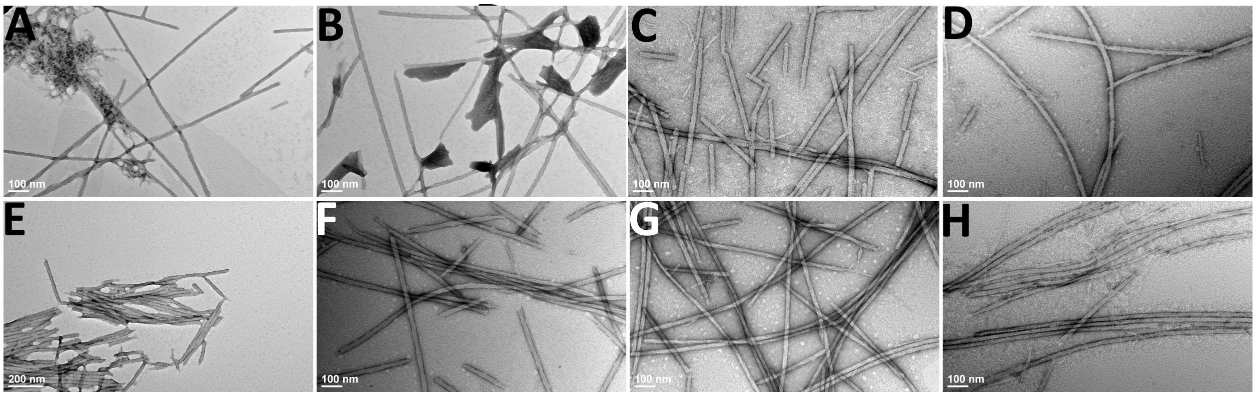

Figure S11. TEM images of Fmoc-F_5-Phe-DAP hydrogels. NaCl gels loaded with **(A)** RNase A, **(B)** TI, **(C)** BSA, **(D)**, human IgG. DMEM gels loaded with **(E)** RNase A, **(F)** TI, **(G)** BSA, and **(H)** human IgG.

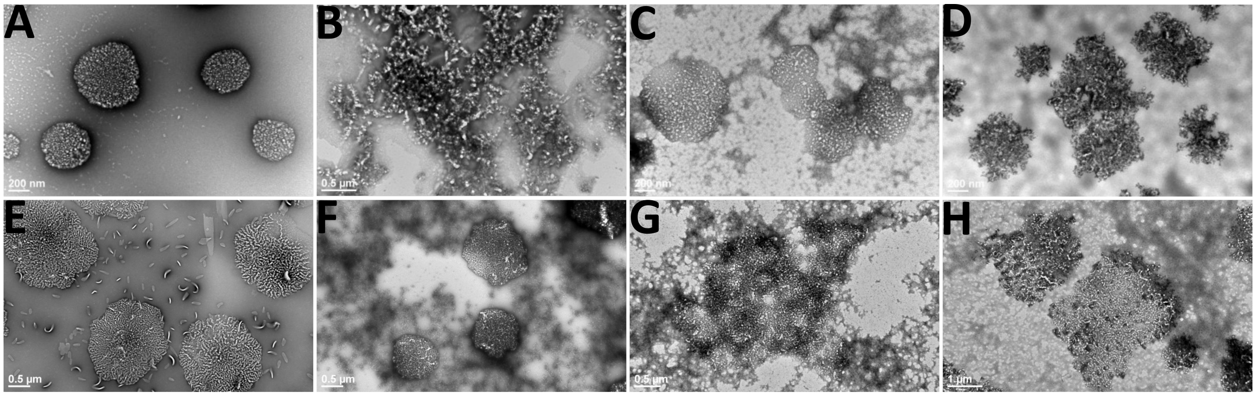

Figure S12. TEM images of model proteins. NaCl solutions containing (A) RNase A, (B) TI, (C) BSA, and (D) Human IgG. DMEM solutions containing (E) RNase A, (F) TI, (G) BSA, and (H) Human IgG.

Table S2. Fibril width measurements for all systems with and without proteins loaded

Protein	NaCl Gels Fibril width (nm)	DMEM Gels Fibril width (nm)
None	23.7 ± 3.3	22.3± 3.6
RNase A	15.4 ± 2.0	18.5 ± 2.5
TI	18.9 ± 2.7	22.5 ± 3.5
BSA	24.9 ± 3.5	20.1 ± 2.5
Human IgG	18.8 ± 2.3	20.7 ± 2.7
Average	**20.6 ± 2.9**	

Table S3. The normalized charge density of each system was calculated by multiplying the net charge by the molar concentration of each protein.

		NaCl Gels		DMEM Gels	
**Protein**	**Molarity (µM)**	**Charge (pH 5)**	**Normalized charge density (µM)**	**Charge (pH 7)**	**Normalized charge density (µM)**
None	-	-		-	-
RNase A	73	18.4	1343.2	12.9	941.7
TI	50	1.2	60	-7.6	-380
BSA	15	23.5	352.5	-10.0	-150
Human IgG	7	54.2 ± 5.8	379.4	-1.1 ± 4.1	-7.7

Table S4. Calculated mesh size for each hydrogel. The molecular weight and charge of each protein are also shown for comparison purposes with the % protein released and the diffusion coefficient.

	Protein	G′ (Pa)	Mesh size (nm)	MW (kDa)	Charge	Total Protein Released (%)	Diffusion Coefficient
**NaCl Gels**	None	2782	11.6	-	-	-	-
	RNase A	1479	14.3	13.7	18.3	22	1.99×10^{-12}
	TI	6057	9.0	20.1	1.1	14.1	7.25×10^{-12}
	BSA	1179	15.4	66.4	23.4	8	1.69×10^{-13}
	Human IgG	885	17.0	150	54.2	5.2	2.94×10^{-14}
**DMEM Gels**	None	196	28.1	-	-	-	-
	RNase A	300	24.4	13.7	12.8	5.6	2.26×10^{-13}
	TI	7950	8.2	20.1	-7.5	0.31	-
	BSA	1426	14.5	66.4	-9.9	3.5	4.83×10^{-15}
	Human IgG	842	17.3	150	-1.1	5.3	2.21×10^{-14}

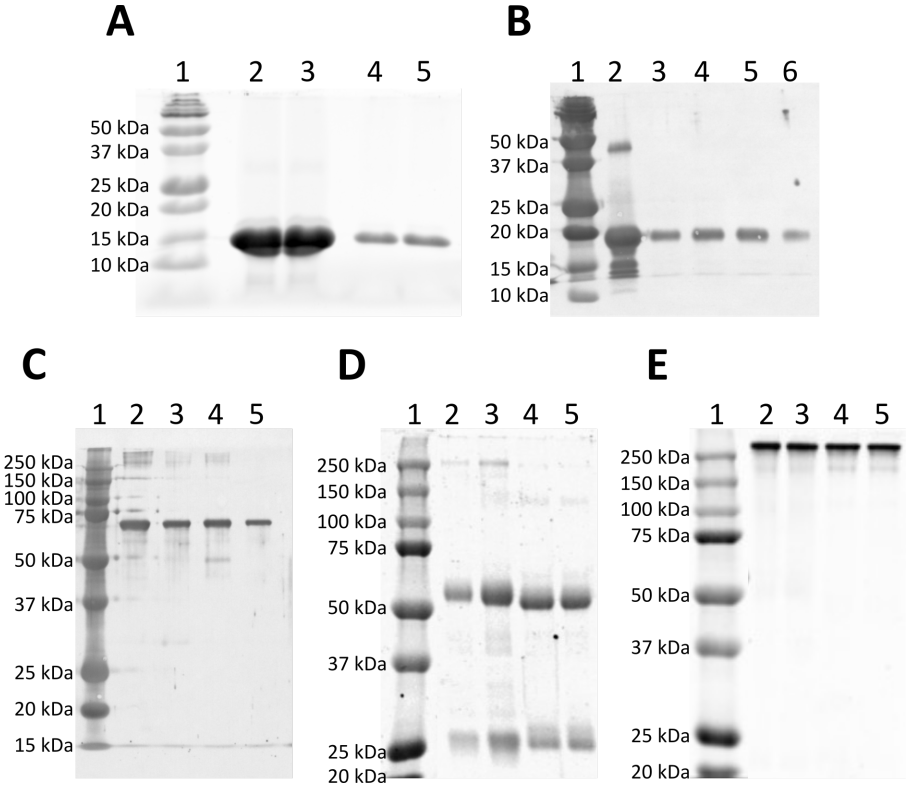

Figure S13. SDS-PAGE of native and released proteins. **(A)** RNase A. Lanes contain (1) Bio-Rad Precision Plus Dual Color Protein Standard, (2-3) native RNase A, and (4-5) released RNase A. **(B)** Western Blot of TI. Lanes contain (1) Bio-Rad Precision Plus Dual Color Protein Standard, (2) native TI, and (3-6) released TI. **(C)** Western blot of BSA. Lanes contain (1) Bio-Rad Precision Plus Dual Color Protein Standard, (2) native BSA, and (3-5) released BSA. **(D)** SDS-PAGE of Human IgG in the presence of β-mercaptoethanol. Lanes contain (1) Bio-Rad Precision Plus Dual Color Protein Standard, (2) native Human IgG, and (3-5) released Human IgG. Image was cropped to remove unrelated samples and place human IgG lanes close to reference ladder. **(E)** SDS-PAGE of Human IgG in the absence of β-mercaptoethanol. Lanes contain (1) Bio-Rad Precision Plus Dual Color Protein Standard, (2) native Human IgG, and (3-5) released Human IgG. Image was cropped to remove unrelated samples and place human IgG lanes close to reference ladder.

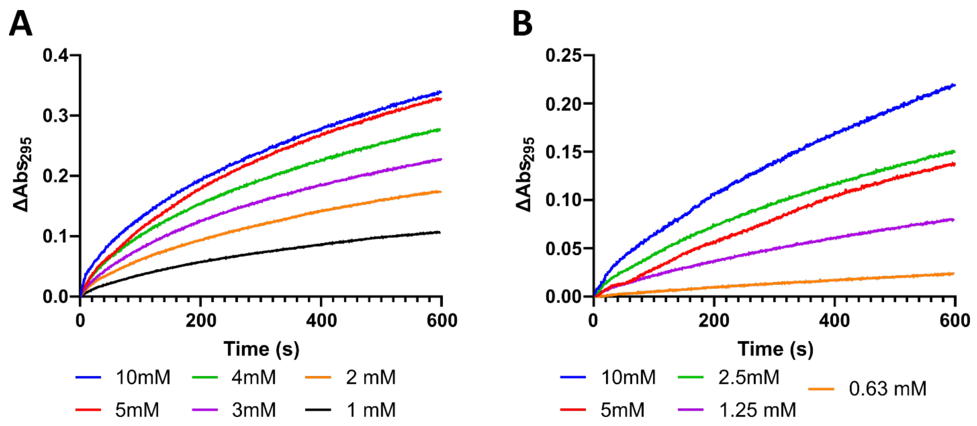

Figure S14. Change in absorbance (295 nm) vs time (s) of **(A)** native and **(B)** released RNase A incubated with varying concentrations of cCMP.

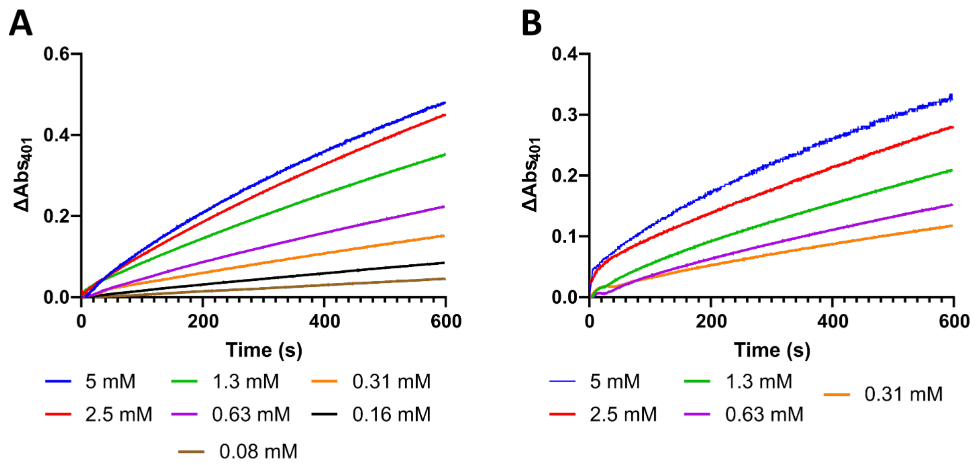

Figure S15. Change in absorbance (401 nm) vs time (s) of **(A)** native and **(B)** released BSA incubated with varying concentrations of *p*-nitrophenyl acetate.
